# Supplementary material for: Efficient ssODN-Mediated Targeting by Avoiding Cellular Inhibitory RNAs through Precomplexed CRISPR-Cas9/sgRNA Ribonucleoprotein
Source: Stem Cell Reports. 2021 Mar 11;16(4):985–96. doi: 10.1016/j.stemcr.2021.02.013 (PMC8072016; doi:10.1016/j.stemcr.2021.02.013)
Supplement: Document S1. Supplemental Experimental Procedures, Figures S1–S5, and Tables S1–S4 [file mmc1.pdf]

Stem Cell Reports, Volume *16*

Supplemental Information

Efficient ssODN-Mediated Targeting by Avoiding Cellular Inhibitory RNAs through Precomplexed CRISPR-Cas9/sgRNA Ribonucleoprotein

Akihiro Kagita, Mandy S.Y. Lung, Huaigeng Xu, Yuto Kita, Noriko Sasakawa, Takahiro Iguchi, Miyuki Ono, Xiou H. Wang, Peter Gee, and Akitsu Hotta

Experimental Procedures

In vitro cleavage assay to measure Cas9's nuclease activity

For one sample, 50 ng recombinant SpCas9 (PNA Bio, Cat. No. CP01; IDT, Cat. No. 1081058; or Thermo Fisher, Cat. No. A36498) was mixed by gentle pipetting with 12.5 ng in vitro transcribed DMD1 sgRNA and 10 ng of a 703 bp PCR amplicon from the human *DMD* gene locus in a total volume of 10 to 20 µL cleavage buffer containing a final concentration of 20 mM HEPES (pH 7.5), 150 mM KCl, 1 mM DTT, 1 mM MgCl2, 50 µg/mL BSA and 10% glycerol and incubated at 37°C for 1 hour. The samples were then treated with 40 ng RNase A at 37°C for 30 minutes and 10 µg Proteinase K at 50°C for 20 minutes to remove residual RNA and protein before the visualization of cleaved DNA bands using the Agilent 2200 TapeStation system.

Intracellular Cas9 activity and quantification measurements by various transfection methods

For plasmid DNA transfection, HEK293T cells were seeded into 6-well plates and transfected with plasmid DNA vectors pHL-EF1a-hcSpCas9-A (2 µg, Addgene #60599) and pHL-H1-sgRNA-EF1a-RiH (2 µg, Addgene #60601) by Lipofectamine 2000 (20 µL). 48 hours after the transfection, bulk cells were harvested for Western blot and T7EI analyses. For Cas9 protein and gRNA transfection, HEK293T cells were seeded into 6-well plates and transfected with either Cas9 protein pre-mixed with sgRNA (RNP pre-mix) or Cas9 protein/sgRNA separately (RNP post-mix) by using Lipofectamine CRISPRMAX. The bulk cells were harvested 4 hours after transfection for Western blot analysis and at 48 hours after the transfection for T7EI analysis. For CRONUS piggyBac vector with Dox/Dex induction, previously established CRONUS-HEK293T cells (Ishida et al., 2018) were seeded into a 6-well plate with (CRONUS[+]) or without (CRONUS[−]) doxycycline (Dox, 2 µM) and dexamethasone (Dex, 1 µM). After 48 hours of the Dox and Dex treatment, the bulk cells were harvested for western blot and T7EI analyses. Note, the leaky expression of CRONUS-Cas9 might be due to the use of FBS, which was not tetracycline-free.

Western blotting

Whole cell pellets were lysed with Lysis buffer (20 mM Tris-HCl, pH=7.5, 150 mM NaCl, 1 mM EDTA, 1% Triton-X) and supplemented with cOmplete protease inhibitor cocktail (Merck, 11873580001) for 15 min and centrifuged (14 000 rpm for 10 min at 4ºC) to remove the insoluble fraction. Total protein concentration was measured by the Bradford assay (BioRad, Cat No. 5000001JA) using a BSA standard curve. After mixing with Sample buffer with 200 mM DTT and denatured with 98ºC for 5 min, we loaded 0.2-0.5 µg total protein per lane on 4-12% gradient gel (GeneScript) for SDS-PAGE in MOPS buffer. After electrophoresis (150 V, 80 mA, 60 min), the gel was equilibrated with 10% methanol in 1× blotting buffer for 10 min. The protein samples were blotted onto a nitrocellulose membrane by using the iBlot Dry Blotting system with P0 program (ThermoFisher). After washing the blotted nitrocellulose membrane with PBS-T for 5 min, the blot was incubated with Blocking One blocking solution (Nacalai tesque) for 1 hour at room temperature.

Streptococcus pyogenes Cas9 protein (160 kDa) was detected with anti-Cas9 mouse monoclonal antibody (Active motif, clone 7A9-3A3, 1:1000 dilution) in 2 mL Can Get Signal 1 solution (Toyobo, NKB-101) and incubated overnight at 4ºC. After washing by PBS-T 3 times, bands were detected with HRP linked anti-mouse IgG secondary antibody (Cell Signaling Technology, #7076, 1:1000 dilution) in 2 mL Can Get Signal 2 solution (Toyobo) for 1 hour at room temperature and imaged with Amersham ECL Western Blot Detection Reagent (GE Healthcare) by using the ChemiDoc XRS+ Imaging system (BioRad). Then, the same membrane was washed with PBS-T two times and stripped with Restore PLUS Western Blot Stripping buffer (ThermFisher, Cat No. 46430) for 30 min at 37ºC. After washing with PBS-T 2-3 times, the membrane was incubated with BlockOne blocking solution for 1 hour at room temperature. As a cell lysate loading control, ACTB (β-actin, 42 kDa) protein was detected with anti-ACTB mouse monoclonal antibody (Sigma-Aldrich, clone AC-15, 1:2000 dilution) in 2 mL Can Get Signal 1 solution and incubated overnight at 4ºC. After washing by PBS-T 3 times, bands were detected with HRP-linked anti-mouse IgG secondly antibody (1:1000 dilution) in 2 mL Can Get Signal 2 solution for 1 hour at room temperature and imaged with Amersham ECL Western Blot Detection Reagent by using the ChemiDoc XRS+ Imaging system.

T7E1 assay

Extracted genomic DNA (100 ng) was used as a template for PCR amplification of the genomic region of interest using PrimeSTAR GXL DNA polymerase (Toyobo, Cat. No. R050A) and primers in **Supplemental Table 1** The resulting PCR amplicon was column purified with a Wizard SV Gel and PCR Clean-Up System (Promega, Cat. No. A9285). Purified PCR product (400 ng) was denatured (95°C for 5 min) and reannealed by gradually cooling from 95°C to 85°C at −2°C/s and 85°C to 2°C at −0.1°C/s in NEB Buffer 2.1 (New England Biolabs) on a thermocycler. After treatment with T7 Endonuclease I (T7E1; New England Biolabs) for 15 min at 37°C, 250 mM EDTA was added at a final concentration of 6 mM to stop the T7EI reaction, and cleaved DNA fragments were analyzed using High Sensitivity D1000 ScreenTapes on a 2200 TapeStation (Agilent Technologies).

In vitro transcription for sgRNA and mRNA preparation

For the *in vitro* transcription of sgRNA, the PCR template was amplified by the KOD Plus Neo PCR Kit (Toyobo, Cat. No. KOD-401) with a forward primer, which contained a T7 promoter, as shown in **Supplemental Table 2**. Then, sgRNA was in vitro transcribed overnight at 37°C using a MEGAshortscript T7 Transcription Kit (ThermoFisher Cat. No. AM1354) from the PCR template, according to the manufacturer's instructions. RNA purification was performed the following day using a RNeasy MinElute Kit (Qiagen, Cat. No. 74204). For the RNA size-dependent inhibition assay, RNA of less than 500 bp in length were prepared using a MEGAshortscript T7 Transcription Kit, and greater than 500 bp in length were produced using the MEGAscript T7 Transcription Kit (ThermoFisher Cat. No. AM1333). The RNA fragments used are listed in **Supplemental Table 3**.

RNA pull-down assay

For preparation of antibody-coated Dynabeads, 10 μL Dynabeads Protein G (Invitrogen, Cat. No. 10003D) was first incubated in 200 μL PBS + 0.05% Tween with anti-HA-probe (F-7) antibody (Santa Cruz, sc-7392) at a concentration of 1 ng/μL, in a 1.5 mL tube for 30 minutes at room temperature on a rotator. At the end of the incubation, the supernatant was removed using a magnetic stand, and the antibody-coated beads were resuspended in 200 μL PBS + 0.05% Tween for a rinse with gentle pipetting. As a sample, 200 ng rCas9-HA, 50 ng DMD1 sgRNA and 4000 ng total cellular RNA extracted from 293T cells were then combined and made up to 50 μL in a protein buffer containing a final concentration of 20 mM HEPES (pH 7.5), 150 mM KCl, 1 mM DTT and 10% glycerol. As 10% input control for the qPCR analysis downstream, 5 μL of these mixtures were kept aside, and the remaining 45 μL were made up to 300 μL with protein buffer, added to the antibody-coated Dynabeads, and then incubated for 30 minutes at room temperature on a rotator. After removal of the supernatant, the beads bound with RNA/protein were washed 3 times with 200 μL protein buffer and resuspended in 100 μL protein buffer in a new 1.5 mL tube. The supernatant was again removed, and the beads were resuspended in 20 μL of 1 mM Tris (pH 8) and incubated at 65°C for 10 minutes to release the RNA components that would have been bounded to Cas9 protein. The supernatant was collected and used for reverse transcription to produce a cDNA template for qPCR. 5 μL of the supernatant was first incubated at 65°C for 5 minutes to denature any possible secondary structure formed by RNA, then left on ice until use. Reverse transcription was performed using the ReverTra Ace qPCR RT Master Mix (Toyobo, FSQ-201) by mixing 2 μL of 5× RT master mix with 5 μL supernatant, made up to 10 μL with Milli-Q, and then incubated at 37°C for 15 minutes, 50°C for 5 minutes, 98°C for 5 minutes. qPCR was then performed by mixing 1 μL of the reverse-transcribed product with 5 pmol of each of the forward and reverse qPCR primers, 10 μL SYBR Select Master Mix (Applied Biosystems, #4472908), and Milli-Q water to 20 μL total volume. StepOne Real-time PCR system (Applied Biosystems) with standard cycling conditions (Preheating of 95ºC for 10 min, 40 cycles of 95ºC for 15 sec and 60ºC for 1 min) and melting curve conditions described in manufacturer's protocol were used. The amount of sgRNA detected in the samples of the supernatant from the RNA-protein pull-down assay was calculated relative to the positive control sample (sgRNA only with no inhibitory RNA added) using the delta Ct method.

Pluripotency marker flow cytometry analysis

iPSCs and HEK293T cells were trypsinized and suspended in PBS with 5% FBS. Single-cell suspensions were stained for the pluripotent stem cell markers SSEA-4 (Alexa Fluor 647 Mouse anti-SSEA-4, BD Biosciences, Cat. No. 560796) and TRA-1-60 (Alexa Fluor 488 Mouse anti-Human TRA-1-60 Antigen, BD Biosciences, Cat. No. 560173) with an antibody dilution rate of 1:100. No antibody staining and single antibody staining controls were also prepared. After filtering through a 70-μm-pore nylon mesh, stained cells were analyzed by flow cytometry using a BD LSRII Fortessa with BD FACSDiva software (BD Biosciences). Cell debris was excluded from the analysis by using forward- and side-scatter gating. No antibody controls were used to adjust the fluorescence detection range, and single antibody staining controls were used to compensate the fluorescence leakage between Alexa Fluor 488 and 647. The data obtained were analyzed with FlowJo software (Tree Star).

Statistical analysis

For comparison between two groups, we used Welch's t-test calculated by the T.TEST function in Excel software with two tailed, non-paired, unequal variance analysis options. For comparison of three groups or more with a single parameter, one-way ANOVA followed by Tukey's multiple comparisons test in GraphPad Prism software was used. For comparison of several groups with a control, one-way ANOVA followed by Dunnett's multiple comparisons test in Prism software was used. For comparison of several groups with two parameters, two-way ANOVA followed by Sidak’s multiple comparisons test in Prism software was used. $p < 0.05$ was considered statistically significant.

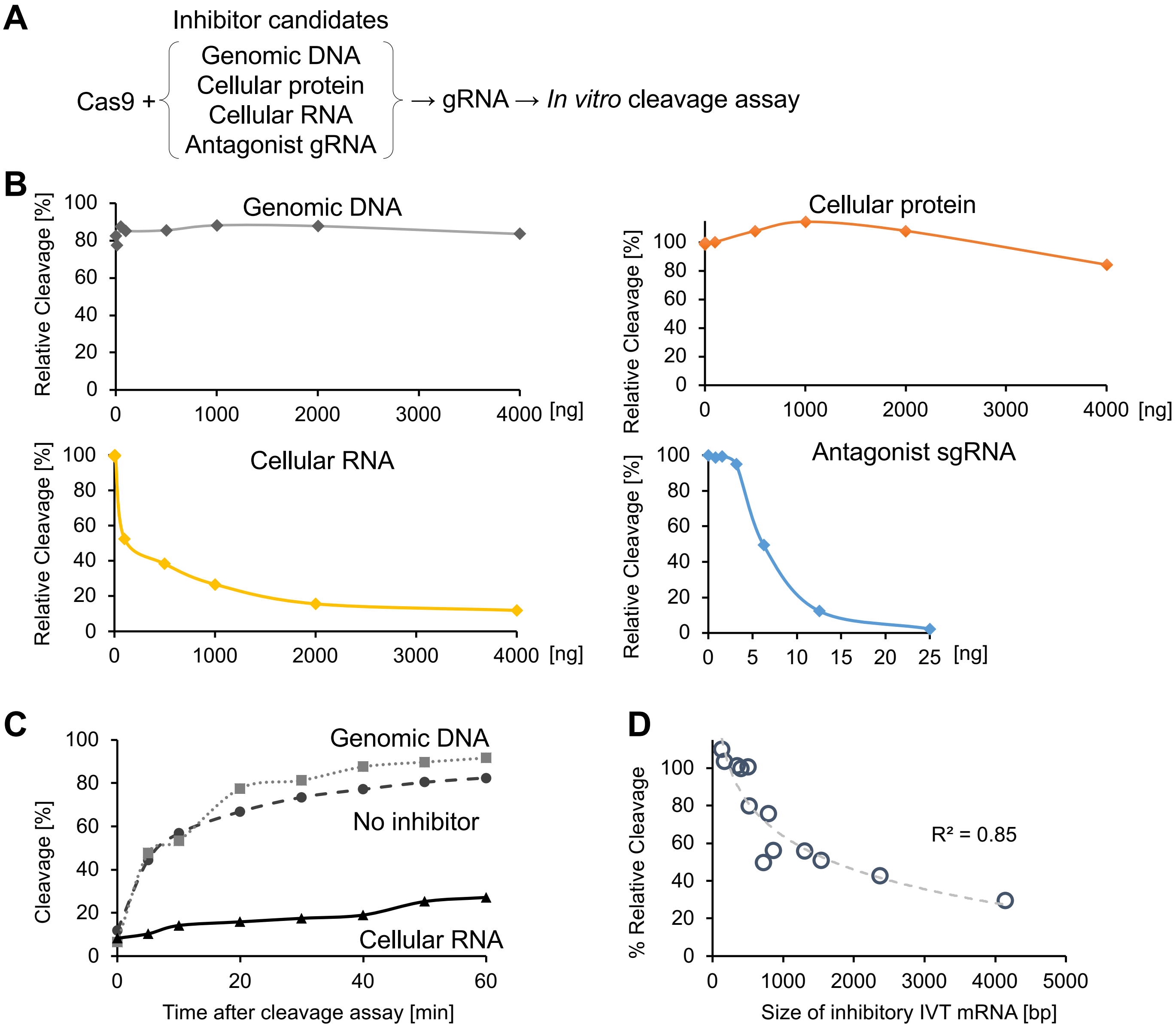

Supplemental Figure 1. Inhibition of Cas9 by cellular RNA and antagonist sgRNA, related to Figure 1.

(A) To examine the inhibitory effect of various cellular components on Cas9 cleavage activity, 50 ng of Cas9 protein was incubated with one of the potential inhibitory factors for 15 minutes at room temperature before the addition of 12.5 ng sgRNA, which targeted human DMD sequence. *In vitro* cleavage reaction was then carried out as described in the Methods section.

(B) To elucidate the effect of various cellular components on Cas9 cleavage activity, we performed an in vitro cleavage assay by first preincubating Cas9 protein with mouse genomic DNA extracted from C2C12 cells (no sgRNA target site), cellular protein and total RNA extracted from HEK293T cells or antagonist sgRNA which targets the HLA-A locus. No apparent inhibition was observed from genomic DNA and cellular proteins, whereas upon preincubation with total RNA relative cleavage was down to 12% at the highest RNA concentration. Also, antagonist sgRNA inhibited relative cleavage activity down to 10% at an equal amount with the targeting DMD sgRNA (12.5 ng), suggesting that sgRNA exchange after binding with Cas9 is not prominent. (C) A time course experiment was performed to elucidate the kinetics of inhibitory effects exerted by genomic DNA and total RNA on Cas9 cleavage activity. We performed an in vitro cleavage assay by first preincubating rCas9 protein with water (negative control), 1,200 ng of genomic DNA or 1,200 ng of total RNA. Samples were collected at 0, 5, 10, 20, 30, 40, 50 and 60 minutes after the beginning of cleavage reaction, and cleavage reaction was stopped by the addition of 1 μL of 0.25 M EDTA and then left on ice until the time course was completed. Results showed that inhibition from total RNA commenced instantaneously and continued throughout the time course.

(D) To examine what type of RNA inhibited on Cas9 cleavage activity, we synthesized various RNAs as shown in Table 2 by *in vitro* transcription and preincubated with Cas9. Results showed that the inhibition on Cas9 cleavage activity was correlated with the size of RNA examined ($R^2 = 0.85$).

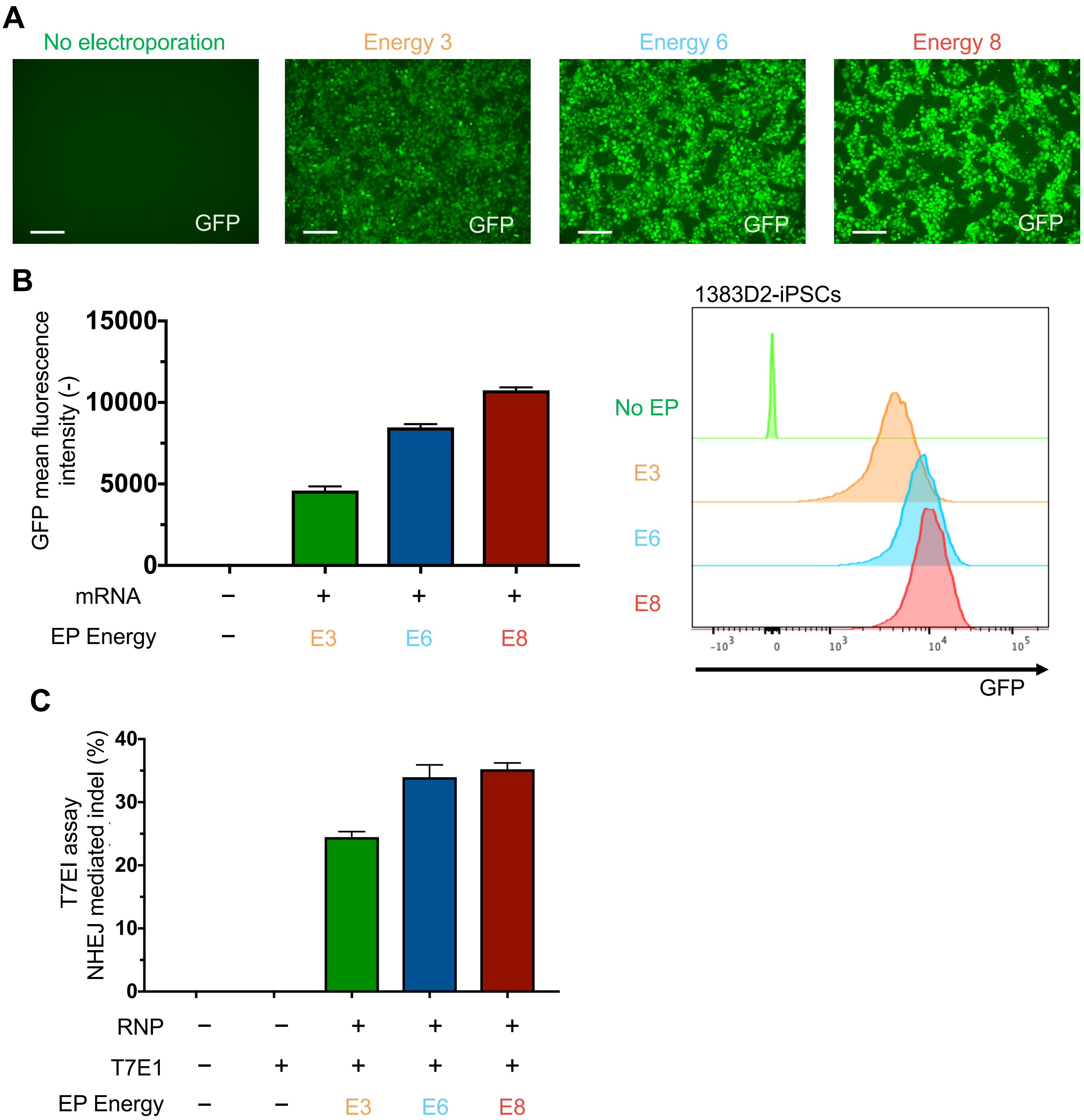

Supplemental Figure 2. Optimization of MaxCyte electroporation, related to Figure 2-5.
(A) 1383D2 human iPSCs were electroporated with GFP mRNA at three electroporation energies (E3, E6, and E8) using MaxCyte ATX. Fluorescence images obtained one day after electroporation are shown. Note that nearly 100% of cells are transfected. There was a modest decrease in cell number at higher energy conditions. Scale bar, 200 μm.
(B) Flow cytometry data of GFP fluorescence intensities are depicted in a bar graph (left panel) or in a half offset overlay plot (right panel) using FlowJo software. The GFP fluorescence signals are presented as mean ± SD from triplicate electroporations.
(C) Pre-complexed Cas9 protein and sgRNA targeting DMD exon 45 was electroporated into 1383D2 iPSCs at three electroporation energies (E3, E6, and E8). Indel efficiencies are presented as mean ± SD from three T7EI assays.

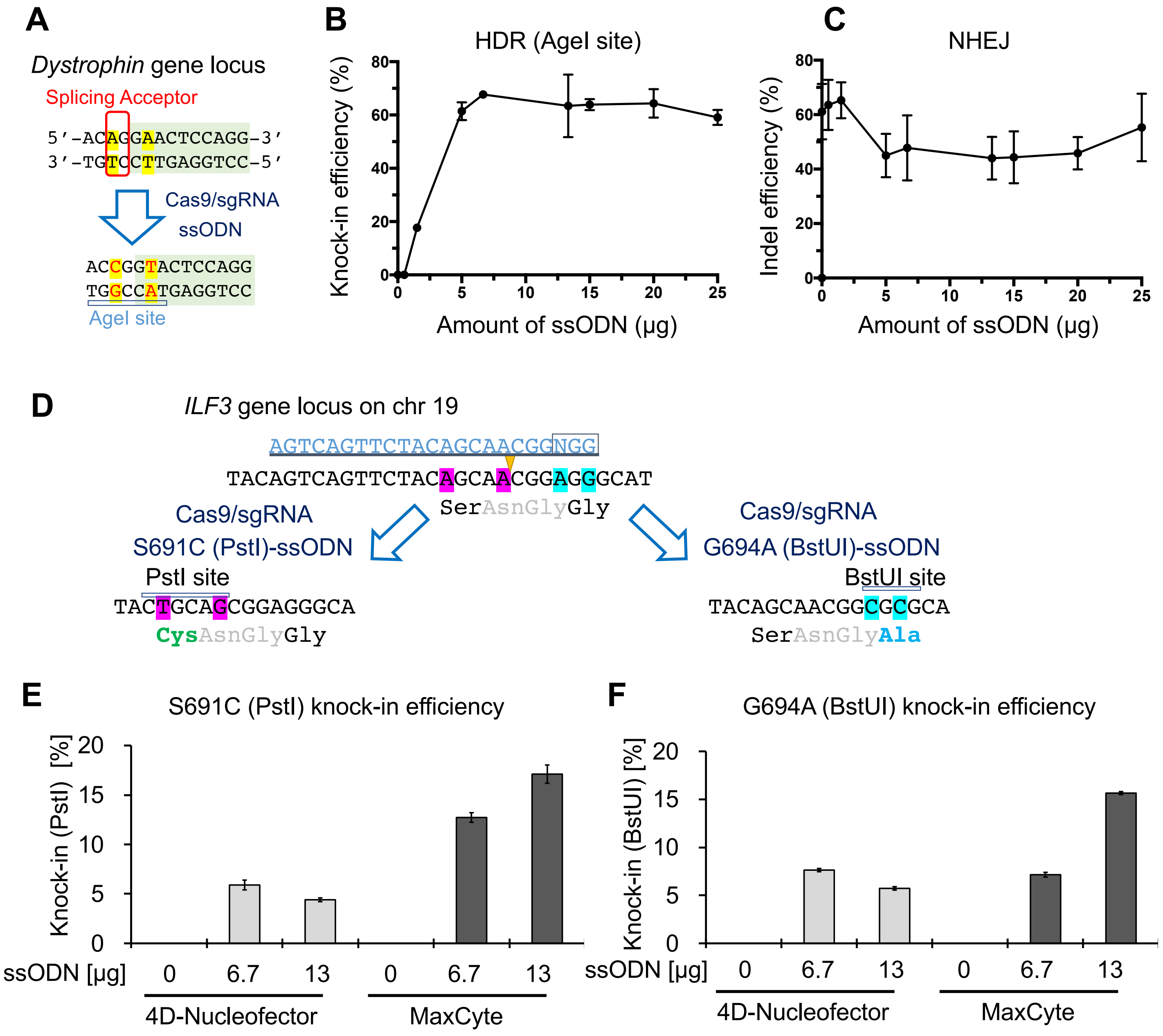

Supplemental Figure 3. Optimization of ssODN and Cas9 RNP mediated targeting, related to Figure 2, 3.

(A) To introduce a mutation at the splicing acceptor of DMD exon 45, we designed an ssODN donor template to replace two nucleotides. Successful knock-in event introduces an AgeI restriction enzyme site (A|CCGGT).

(B) Knock-in efficiency at the *DMD* gene locus in DMD-iPSCs (CiRA00111) was assessed by AgeI restriction enzyme digest, when various amounts of ssODN were electroporated by MaxCyte with energy E8. Scatter plot of ssODN knock-in frequency quantified from three AgeI digestion experiments are represented as mean ± SD (n = 3).

(C) Scatter plot of NHEJ mediated indel frequency measured by three T7EI assays are represented as mean ± SD (n = 3).

(D) Schematic of genomic DNA sequences at the *ILF3* (*NF110*) gene locus on chromosome 19, representing the introduction of Ser671Cys and Gly694Ala substitution with ssODN-mediated knock-in (KI). Successful knock-in sequence generates a PstI or BstUI restriction enzyme site, respectively.

(E) With a healthy donor derived human iPSCs (1383D2 clone), ssODN-mediated genome editing was performed with various ssODN amounts and two electroporation instruments. The knock-in efficiency was evaluated based on the restriction enzyme digestion assay by using PstI enzyme (recognition site: CTGCA|G). PstI knock-in percentages are represented as mean ± SD (n = 3, technical triplicate).

(F) Similar to (B), ssODN-mediated knock-in efficiency was evaluated by the restriction enzyme digestion assay with BstUI enzyme (recognition site: CG|CG). BstUI knock-in efficiencies are represented as mean ± SD (n = 3, technical triplicate).

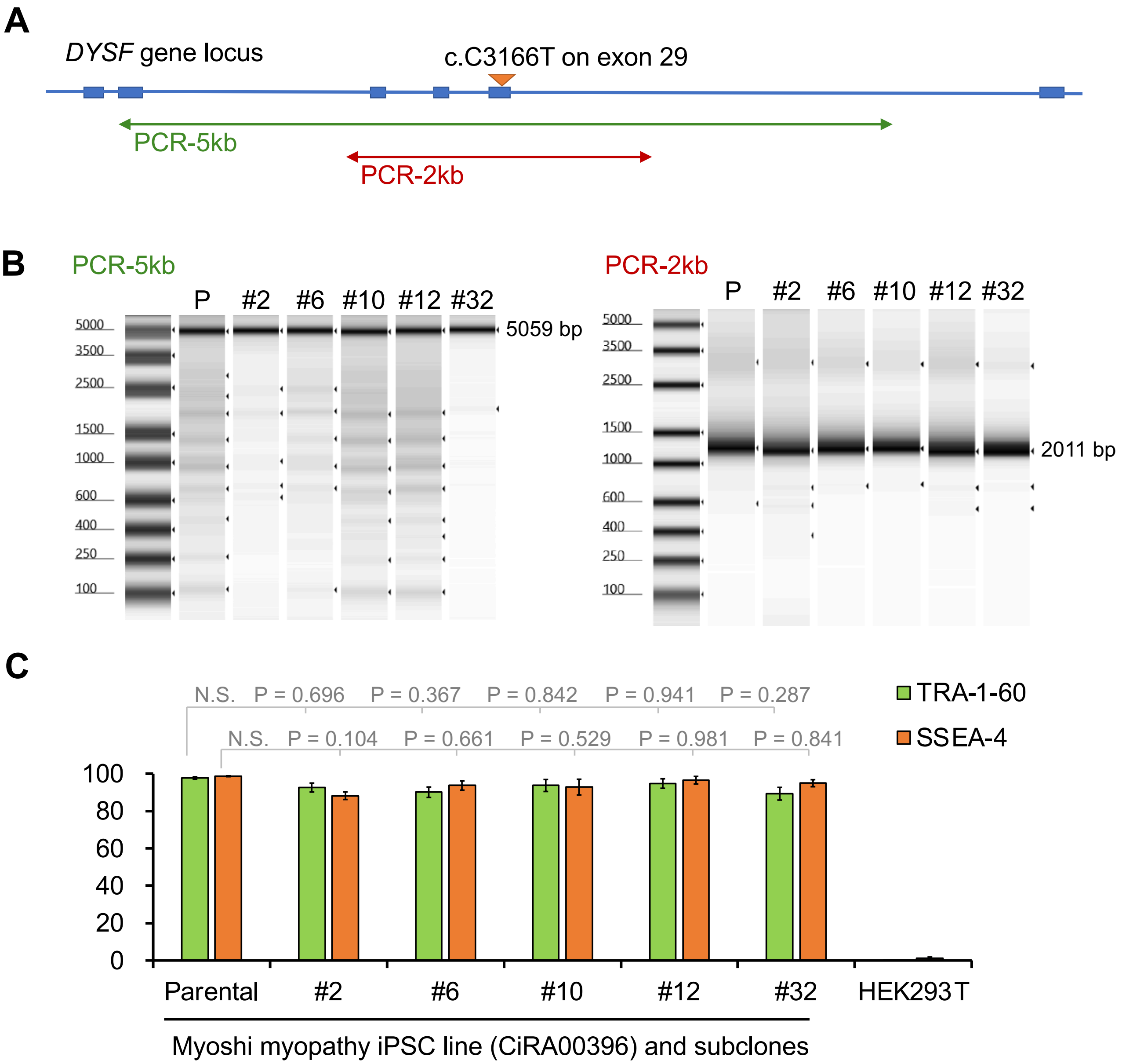

Supplemental Figure 4. Analysis of on-target large deletion and pluripotency, related to Figure 2.
(A) Schematic of the *DYSF* gene locus around the target c.C3166T homozygous mutation site on exon 29. To check whether there an on-target large deletion exists, we designed two primer pairs to amplify 5 kb or 2 kb regions, respectively.
(B) Genomic DNA was extracted from the parental Myoshi myopathy patient iPSC line (CiRA00396) and genome edited subclones that were homozygously (clones #2, #10, #12, and #32) or heterozygously (clone #6) corrected by Cas9/sgRNA using MaxCyte. All 5 clones showed no apparent deletion within the analyzed region.
(C) The above subclones were stained for cell surface TRA-1-60 and SSEA-4 as pluripotent stem cell markers and analyzed by flow cytometer. HEK293T cells were used as negative control. The levels of pluripotency marker expression with the 5 subclones were comparable with the parental iPSC line CiRA00396. Data are represented as mean ± SD (n = 3 experiments). One-way ANOVA followed by Dunnett's test.

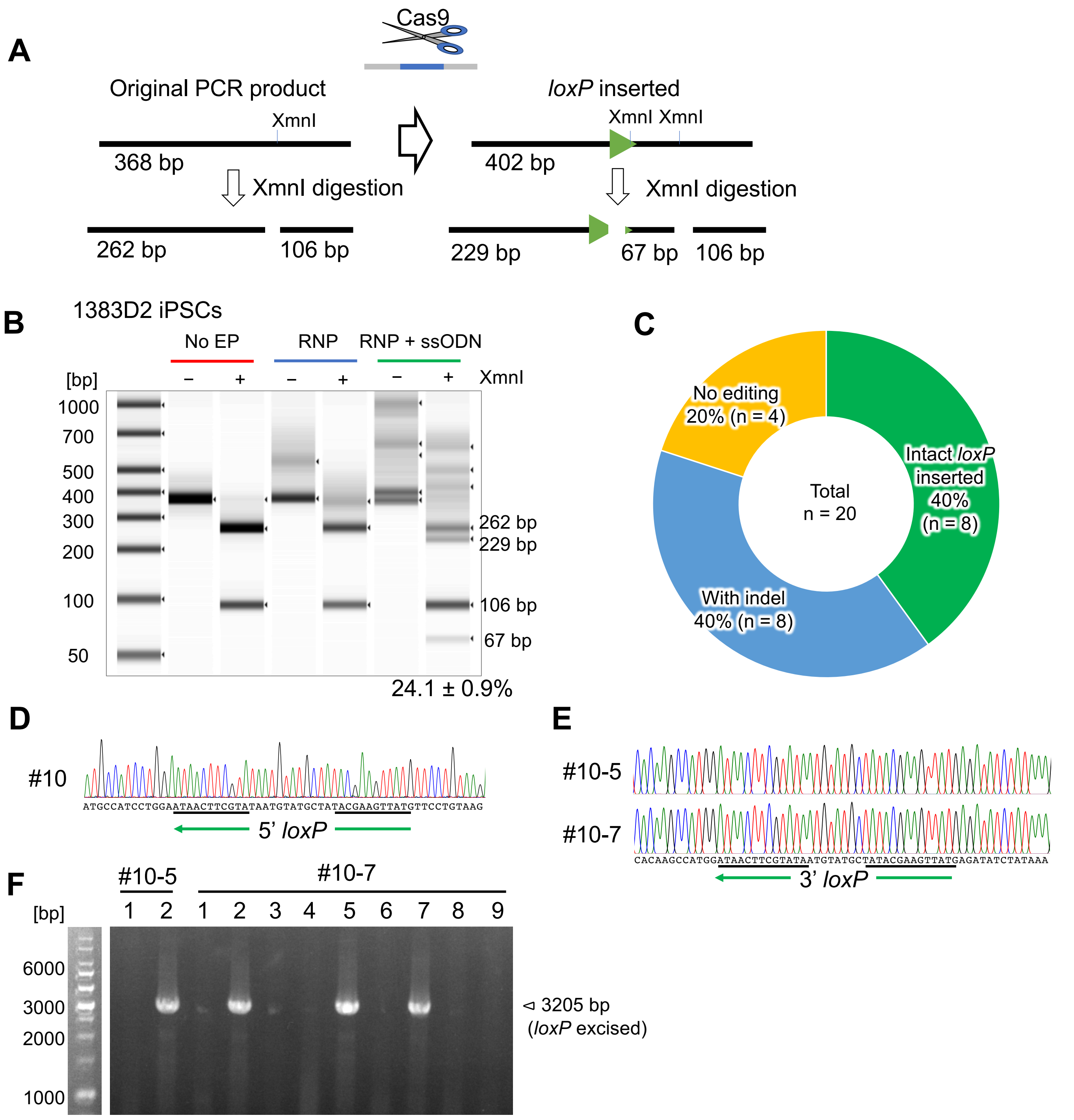

Supplemental Figure 5. Insertion of *loxP* site by ssODN, related to Figure 4.

(A) Schematic to insert a *loxP* sequence at the *DMD* gene locus in 1383D2 iPSCs. Successful insertion of a *loxP* site generates an extra XmnI restriction enzyme site.

(B) TapeStation analysis of PCR products from 1383D2 iPSCs with or without XmnI digestion. With RNP and ssODN electroporation, extra bands with expected sizes can be observed, suggesting the *loxP* sequence is inserted at the target site.

(C) We isolated subclones of 1383D2 iPSCs and genotyped each clone by Sanger sequencing. Pie chart represents 8 subclones out of 20 subclones analyzed have intact *loxP* sequence insertion at the target site.

(D) Sanger sequencing of the 1st *loxP* site in clone #10 (Reverse primer).

(E) Sanger sequencing of the 2nd *loxP* site in clones #10-5 and #10-7 (Reverse primer).

(F) After Cre treatment of clone #10-5 or #10-7, subclones were isolated and genotyped by PCR. Clones #10-5-2, #10-7-2, -5, and -7 showed successful excision of the 342 kb region flanked by the two *loxP* sites.

Supplemental Table 1. PCR and Sanger sequencing primers. Related to Experimental Procedures section.

Name	Sequence
DMD1-qPCR-Fwd	GGTATCTTACAGGAACTCCGT
DMD1-qPCR-Rev	GACTCGGTGCCACTTTTTCAAG
CEL-I-DMD-Fwd	CACCTCTCGTATCCACGATCACTAAG
Ex45 Scr-Rev3 new	TAGTGCCTTTCACCCTGCTTAT
DYSF-in28-L-fwd	AACCTGGCGAGCCCCAT
DYSF-in29-rev	GTGTGATCTGTGCATGCGC
DYSF-ex29-fwd2	ATCACCATCCCCCCGGAG
DYSF-in29-rev2	TGTGGGCATGCAGGTCTG
DYSF_deletion_check_F	GAACGAGACTAAGTTGGCCCTTG
DYSF_deletion_check_R	CAGCTGGTTGTCCAAAAGCC
DYSF_deletion_check2_F	CTGTCTGCCTTGGTCTCTTGCAC
DYSF_deletion_check2_R	CTGGGTGTGATCTGTGTGTGATCTC
T7E1 Fwd new 368bp	CCCTGACACATAAAAGGTGTCTTTCTGT
T7E1 DMD1 Rev	TTCTGTCTGACAGCTGTTTGCAGAC
NF110-ssODN-Fw	TCAGGACCCATGGCTGCCG
NF110-ssODN-Rv	GGGACTGGTAGCCCGAGC
DMDexon55(45-55)check_dir5	CCTCGGGTACACTGAAAGTTATGTG
DMDexon45(45-55)check_rev5	CACCACAGGCTTTAACTTCTGCCG
604B1-HLA-C-be-ex1-fwd	CAATCAGCGTCTCCGCAGT
604B1-HLA-C-ex8-rev	ATGCTAACAGGAACGCAGACA
HLA-C-ex1-fwd	TCCTGCTGCTCTCGGGAG
HLA-C0702-in3-rev	AGGGAGGGCGATATTCCAGT

Supplemental Table 2. Primers used for *in vitro* transcription reaction to prepare sgRNA. Related to Experimental Procedures section.

Name	Sequence
T7-DMD-sgRNA1 fwd	GAAATTAATACGACTCACTATAgggtatcttacaggaactccGTTTTAGAGCTAGAAATAGCAAG
T7-DMD-in55-g3-IVT-fwd	GAAATTAATACGACTCACTATAggactttatagatatctcccaGTTTTAGAGCTAGAAATAGCAAG
T7-DYSF-gRNA2-IVT	GAAATTAATACGACTCACTATAggtactacacacactgacggGTTTTAGAGCTAGAAATAGCAAG
T7-NF110-Ex17-SA2-fwd	GAAATTAATACGACTCACTATAggtcagttctacagcaacggGTTTTAGAGCTAGAAATAGCAAG
T7-HLA-A-0101-ex3-g2-IVT-fwd	GAAATTAATACGACTCACTATAggccgtcgtaggcgtcctgcGTTTTAGAGCTAGAAATAGCAAG
T7-HLA-C7-ex2g2-IVT-fwd	GAAATTAATACGACTCACTATAGgcaggttccgcaggctcactGTTTTAGAGCTAGAAATAGCAAG
sgRNA-+85 rev	AAAGCACCGACTCGGTGCCACTTTTTCAAGTTGATAACGGACTAGCCTTATTTTAACTTGCTATTTCTAGCTCTAAAAC

Supplemental Table 3. RNA fragments for *in vitro* cleavage inhibition assay. Related to Figure S2B.

Source	RNA type	Size (bp)
SpCas9	mRNA	4408
hF8	mRNA	2365
hEEF1G	mRNA	1536
hF8	mRNA	1300
EGFP	mRNA	985
hRPL8	Ribosomal mRNA	856
hF8	mRNA	787
hF8	mRNA	515
hHIST1H2AG	mRNA	498
hF8	mRNA	402
hRPPH1	Non-coding RNA	341
hRNU1-3	Small nuclear RNA	164
FnCas9 sgRNA	Orthogonal sgRNA	154

Supplemental Table 4. Template ssODN sequence for HDR experiments. Related to Experimental Procedures section.

Name	Sequence
DMD1 (100-mer)	AAAAAGACATGGGGCTTCATTTTTGTTTTGCCTTTTTGGTATCTTACCGGTACTCCAGGATGGCATTGGGCAGCGGCAAACTGTTGTCAGAACATTGAAT
DYSF-WT-ssODN (100-mer)	GGAAGCCGAAGCACTGGGTCCCTGCTGAGAAGATGTACTACACACACCGACGGCGGCGCTGGGTGCGCCTGCGCAGGAGGGATCTCAGCCAAATGGAAGC
NF110-BstUI-dPAM-ssODN (100-mer)	GCCTGACCCACTGCCTCCCTGTTTAGGTCAGTTCTACAGCAACGGCGCGCATTCTGGGAATGCCAGTGGCGGTGGCGGCGGGGGCGGTGGTGGCTCCTCC
NF110-PstI-ssODN (100-mer)	GCCTGACCCACTGCCTCCCTGTTTAGGTCAGTTCTACTGCAGCGGAGGGCATTCTGGGAATGCCAGTGGCGGTGGCGGCGGGGGCGGTGGTGGCTCCTCC
DMD1+loxP-ssODN (134-mer)	AAGACATGGGGCTTCATTTTTGTTTTGCCTTTTTGGTATCTTACAGGAACATAACTTCGTATAGCATACATTATACGAAGTTATTCCAGGATGGCATTGGGCAGCGGCAAACTGTTGTCAGAACATTGAATGCA
DMD-in55-g3+loxP-ssODN (134-mer)	TCATTTGGAGGTAATTTGTTTGGAACAGTATCAGACTTTATAGATATCTCATAACTTCGTATAGCATACATTATACGAAGTTATCCATGGCTTGTGATAGAATATAAGGGCAATGCAAATGTAGAGTTTTTTGC
C0702-1bp-mut-AS (100-mer)	CGGGGTCACTCACCGTCCTCGCTCTGGTTGTAGTAGCCGCGCAGGTTCCGCAGaCTCACTCGGTCAGCCTGTGCCTGGCGCTTGTACTTCTGTGTCTCCC
C0702-2bp-mut-AS (100-mer)	CGGGGTCACTCACCGTCCTCGCTCTGGTTGTAGTAGCCGCGCAGcTTCCGCAGGtTCACTCGGTCAGCCTGTGCCTGGCGCTTGTACTTCTGTGTCTCCC
