# Supplementary material for: Efficient ssODN-Mediated Targeting by Avoiding Cellular Inhibitory RNAs through Precomplexed CRISPR-Cas9/sgRNA Ribonucleoprotein
Source: Stem Cell Reports. 2021 Mar 11;16(4):985–96. doi: 10.1016/j.stemcr.2021.02.013 (PMC8072016; doi:10.1016/j.stemcr.2021.02.013)
Supplement: Document S2. Article plus Supplemental information [file mmc2.pdf]

Stem Cell Reports
Resource
OPEN ACCESS

Efficient ssODN-Mediated Targeting by Avoiding Cellular Inhibitory RNAs through Precomplexed CRISPR-Cas9/sgRNA Ribonucleoprotein

Akihiro Kagita,[1,2] Mandy S.Y. Lung,[1,2] Huaigeng Xu,[1,2] Yuto Kita,[1] Noriko Sasakawa,[1] Takahiro Iguchi,[1] Miyuki Ono,[1] Xiou H. Wang,[1] Peter Gee,[1,3] and Akitsu Hotta[1,3,*]
[1]Department of Clinical Application, Center for iPS Cell Research and Application (CiRA), Kyoto University, 53 Shogoin-Kawahara-cho, Sakyo-ku, Kyoto 606-8507, Japan
[2]These authors contributed equally
[3]Co-senior author
*Correspondence: akitsu.hotta@cira.kyoto-u.ac.jp
https://doi.org/10.1016/j.stemcr.2021.02.013

SUMMARY

Combined with CRISPR-Cas9 technology and single-stranded oligodeoxynucleotides (ssODNs), specific single-nucleotide alterations can be introduced into a targeted genomic locus in induced pluripotent stem cells (iPSCs); however, ssODN knockin frequency is low compared with deletion induction. Although several Cas9 transduction methods have been reported, the biochemical behavior of CRISPR-Cas9 nuclease in mammalian cells is yet to be explored. Here, we investigated intrinsic cellular factors that affect Cas9 cleavage activity *in vitro*. We found that intracellular RNA, but not DNA or protein fractions, inhibits Cas9 from binding to single guide RNA (sgRNA) and reduces the enzymatic activity. To prevent this, precomplexing Cas9 and sgRNA before delivery into cells can lead to higher genome editing activity compared with Cas9 overexpression approaches. By optimizing electroporation parameters of precomplexed ribonucleoprotein and ssODN, we achieved efficiencies of single-nucleotide correction as high as 70% and *loxP* insertion up to 40%. Finally, we could replace the HLA-C1 allele with the C2 allele to generate histocompatibility leukocyte antigen custom-edited iPSCs.

INTRODUCTION

Human induced pluripotent stem cells (iPSCs) are important research tools for studying diseases and a promising cell source for regenerative medicine (Takahashi et al., 2007; Takahashi and Yamanaka, 2016). Recent advances in genome editing technologies enable the correction of a pathogenic mutation in patient iPSCs or the introduction of a desired genetic alteration in healthy iPSCs for studying genetic function or modulating cellular characteristics (Hockemeyer and Jaenisch, 2016; Hotta and Yamanaka, 2015). In particular, the CRISPR-associated protein (Cas9) system found in *Streptococcus pyogenes* has become the most widely utilized tool for genome editing (Jinek et al., 2012). Cas9 nuclease forms a complex with crRNA and tracrRNA (or single guide RNA [sgRNA]) to target a specific DNA sequence and induce a double-stranded break (DSB). The DNA is repaired by one of several DNA repair pathways, such as non-homologous end-joining (NHEJ), which introduces insertions/deletions (indels), and homology-directed repair (HDR), which requires a DNA donor template in order to make precise repairs.

Because more than half of human pathogenic mutations are single-nucleotide polymorphisms (SNPs) (Rees and Liu, 2018), the introduction of an SNP into iPSCs is of high importance. Such genetic manipulation can be utilized for correcting a pathogenic mutation or introducing a desired mutation. Among various genome editing tools, such as base editors and prime editing (Anzalone et al., 2020), the single-stranded DNA or single-stranded oligodeoxynucleotide (ssODN)-mediated HDR approach is widely used to perform precise genome editing at a desired locus. Several studies have been previously reported to achieve successful ssODN-mediated HDR in iPSCs by utilizing cell-cycle regulators (Lin et al., 2014; Yang et al., 2016), chemical inhibitors to suppress NHEJ (Ma et al., 2018; Riesenberg and Maricic, 2018; Yu et al., 2015), sib selection (Miyaoka et al., 2014, 2016), optimization of transfection conditions (Li et al., 2016; Okamoto et al., 2019), chemical modification of ssODN molecules (Carlson-Stevermer et al., 2017; Savic et al., 2018), and a stably integrated inducible SpCas9 expression vector (Bertero et al., 2016; Chen et al., 2015; Dow et al., 2015; Ishida et al., 2018), since high levels of SpCas9 expression in target cells are thought to be correlated with high levels of genome editing activity in general.

Recently, CRISPR-Cas9 ribonucleotide protein (RNP) delivery has been reported to achieve efficient indel induction and knockin in iPSCs (Dastidar et al., 2018; Kim et al., 2014; Liang et al., 2015; Martin et al., 2019; Xu et al., 2019), even though the Cas9 protein introduced into the cells is transient (typically less than 24 h). However, to the best of our knowledge, the underlying mechanisms as to why RNP transduction results in higher genome editing efficiency relative to the amount of Cas9 protein (Banan, 2020) are unknown. In addition, the ssODN-mediated knockin approach warrants further optimization and efficiency to allow more flexible and desired genome

This is an open access article under the CC BY license (http://creativecommons.org/licenses/by/4.0/).

editing outcomes, such as one-step biallelic modification and insertion of a *loxP* site, with less labor-intensive clonal isolation and genotyping processes.

In this study, we investigated intrinsic cellular factors that affect the editing efficiency of the CRISPR-Cas9/sgRNA complex and found that cellular RNA inhibits Cas9 nuclease and sgRNA binding *in vitro*. Precomplexing Cas9 protein and sgRNA before delivery into cells was far more efficient at inducing indels compared with DNA plasmid-mediated delivery. Thus, we optimized an RNP-based electroporation approach in patient iPSCs with ssODNs in order to induce high levels of knockin exceeding 70%. When we compared two electroporation instruments, Lonza 4D-Nucleofector and MaxCyte, side by side, we found that the MaxCyte protocol showed a tendency toward higher knockin efficiencies under the conditions used. Furthermore, we could apply our approach to generate various iPSC lines by introducing a desired point mutation, by correcting a homozygous pathogenic mutation, or by inserting *loxP* sites without the need for antibiotic selection. Finally, by introducing two point mutations into the *HLA-C* gene, we generated a custom histocompatibility leukocyte antigen (HLA)-edited iPSC line that could evade immune surveillance from natural killer (NK) cells, suggesting that custom HLA-matching iPSCs can be generated by ssODN-mediated HDR.

RESULTS

Intracellular Cas9 Protein Levels Do Not Correlate with Genome Editing Outcomes

We first evaluated genomic DNA cleavage activity in HEK293T cells by comparing the transfection of various forms of Cas9 and sgRNA: lipofection of plasmid DNA vector, lipofection of Cas9 protein and *in vitro* transcribed (IVT) sgRNA, and stable expression of Cas9 and sgRNA from doxycycline (Dox)- and dexamethasone (Dex)-inducible CRONUS *piggyBac* vector (Ishida et al., 2018). Interestingly, we found that, although RNP transfection resulted in 13-fold lower levels of Cas9 protein compared with plasmid DNA (Figure 1A), the cleavage activity of RNP was 1.5-fold higher than that of plasmid DNA transfection by T7E1 analysis (Figure 1B). Furthermore, when RNP lipofection and the CRONUS *piggyBac* vector-mediated Cas9-inducible expression were compared, cleavage activity was comparable, even though the amount of Cas9 protein in cells was 20-fold more abundant using the CRONUS system. However, the high cleavage activity was compromised when recombinant Cas9 and IVT sgRNA were transfected separately ("RNP separate" in Figure 1B). From these observations, we hypothesized that Cas9 might be inactivated or inhibited by an intracellular molecule in mammalian cells.

Endogenous RNA Potentially Interferes with sgRNA Binding to Cas9

To search for cellular components that may interfere with Cas9 cleavage activity in cells, we performed an *in vitro* cleavage assay using a DNA fragment as a substrate for Cas9/gRNA cleavage. We initially hypothesized that an excess amount of genomic DNA might sequester Cas9 protein, but the addition of genomic DNA prior to sgRNA showed almost no inhibition (Figure S1). We also tested cellular protein fractions from cell lysates and observed no significant inhibition. Interestingly, when total cellular RNA from human cells was preincubated with Cas9 protein before the addition of IVT sgRNA (DMD1), DNA cleavage activity of Cas9 was inhibited in a dose-dependent manner, whereas no inhibition was observed when sgRNA was added prior to cellular RNA (Figure 1C). This suggested that total cellular RNA may have an inhibitory effect by preventing sgRNA from complexing with Cas9 protein. Preincubation with non-targeting sgRNA as an antagonist also strongly reduced the subsequent cleavage activity (Figure S1B), suggesting sgRNA switching is rare once sgRNA binds to Cas9 protein.

A time-course experiment revealed that Cas9 cleavage inhibition from total cellular RNA occurred immediately and was sustained. In contrast, genomic DNA exerted no apparent inhibitory effect, similar to the no-inhibitor negative control (Figure S1C).

Inhibition of Cas9 by Cellular RNA Is Reversible by RNase Treatment

To determine whether the inhibition effect by cellular RNA is reversible, we incubated Cas9 protein with inhibitory RNAs (cellular RNAs or antagonist sgRNA) and then performed RNase treatment to see if removal of the inhibitory RNA component from Cas9 would allow sgRNA to complex again and DNA cleavage to occur. After the addition of RNase inhibitor to protect sgRNA from RNase, we added sgRNA and proceeded with the *in vitro* cleavage assay (Figure 1D). We found that RNase treatment managed to reverse the inhibitory effect of cellular RNA as well as that of antagonist sgRNA. This confirms that oligomeric RNA, not monomeric nucleotides, is a key molecule of the inhibitory effect of Cas9. Interestingly, when we examined the various sizes of IVT RNA on Cas9 activity inhibition, larger RNA fragments showed similar inhibition activity compared with bulk cellular RNA (Figure S1D), suggesting that the inhibition effect is sequence independent, but size dependent.

We also performed an RNA pull-down assay to quantify the amount of sgRNA bound to Cas9 in the presence or absence of inhibitory cellular RNA by qRT-PCR. The results showed that the presence of inhibitory cellular RNA significantly ($p = 0.004$ by Welch's t test) reduced

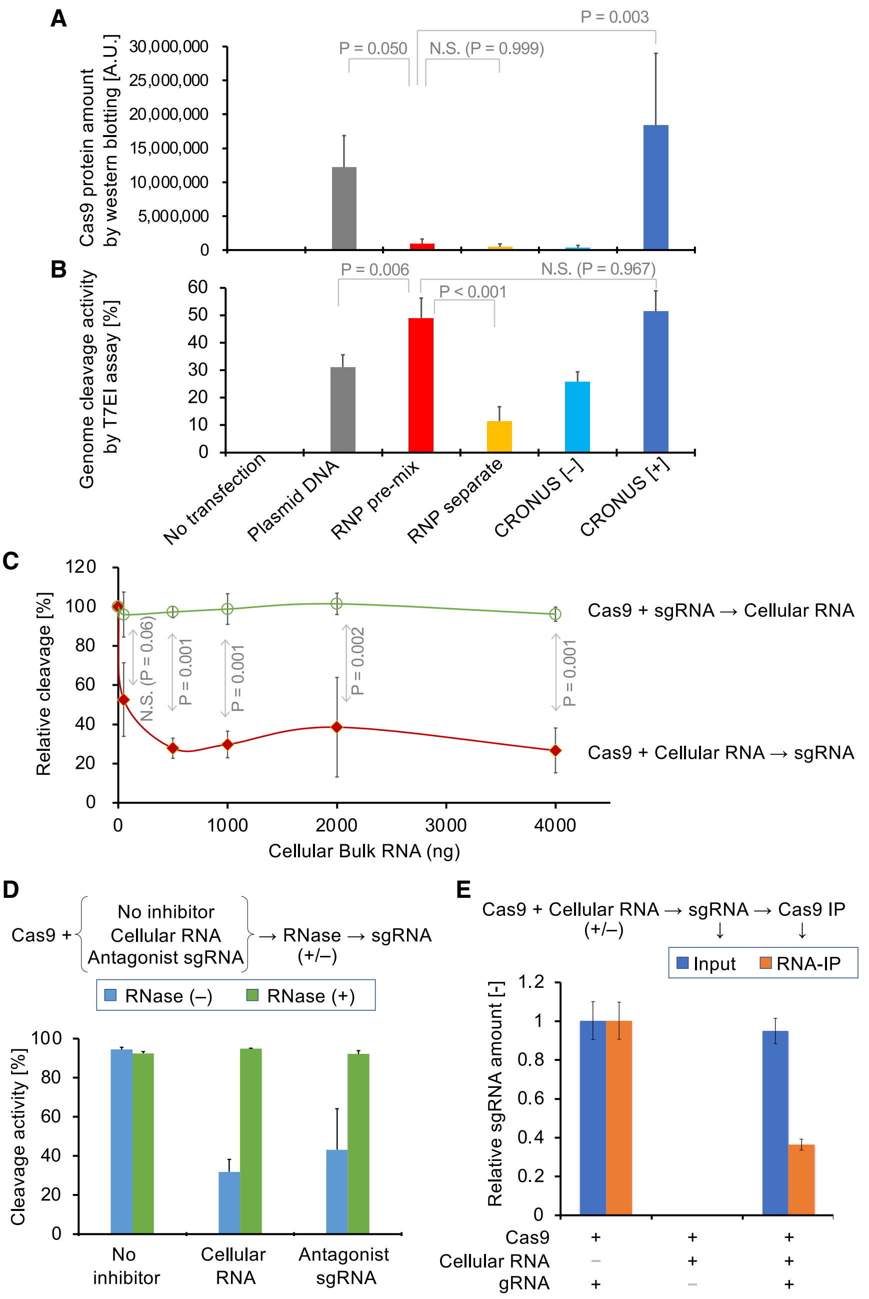

Figure 1. Expressed Cas9 Protein in Cells Can Be Inhibited by Cellular RNA

(A) Intracellular Cas9 protein amount in HEK293T cells was measured by western blot after various transfection methods: transient transfection of plasmid DNA vector by Lipofectamine 2000 (Plasmid DNA, 48 h post-transfection), lipofection of Cas9 protein using Lipofectamine CRISPRMAX (4 h post-transfection) either premixed with sgRNA (RNP pre-mix) or transfected separately (RNP separate), or stably integrated CRONUS *piggyBac* vector with Dox/Dex induction (CRONUS[+], 48 h post-induction) or residual expression (CRONUS [−]). Cas9 band intensities are represented as means ± SD (n = 3 experiments). One-way ANOVA followed by Dunnett's multiple comparisons test.

(B) Genome editing efficiency was measured by T7 endonuclease I (T7EI) assay after 48 h of transfection or induction in (A) at the human *DMD* gene locus. DNA cleavage percentages are represented as means ± SD (n = 3 experiments). One-way ANOVA followed by Dunnett's multiple comparisons test.

(C) Cas9 protein (50 ng) was incubated for 15 min *in vitro* with total cellular RNA (1–4,000 ng) extracted from HEK293T cells before (red line) or after (green line) the addition of sgRNA (12.5 ng). DNA cleavage activity was assessed by the *in vitro* cleavage assay using TapeStation 2200. DNA cleavage percentages are represented as means ± SD (n = 3 experiments). Two-way ANOVA followed by Sidak's multiple comparisons test.

(D) To identify whether the inhibitory effect is dependent on oligomeric RNA and can be reversed, Cas9 protein was preincubated with cellular RNA for 15 min at room temperature. For one group, no RNase A was added (blue bars). For another group, RNase A was added to digest the inhibitory RNA and then RNase inhibitor so as not to interfere with sgRNA, which was added afterward (green bars). Anti-DMD sgRNA was used for target DNA cleavage, and anti-HLA sgRNA was used as a non-cleaving, antagonist sgRNA. *In vitro* DNA cleavage percentage values are presented as means ± SD (n = 3, technical triplicate).

(E) RNA pull-down assay confirmed that cellular RNA interferes with sgRNA binding to Cas9. Recombinant Cas9 protein with a hemagglutinin (HA)-tag was treated with or without cellular RNA before the addition of sgRNA, and the amount of sgRNA was measured by qRT-PCR as input (blue bars). Then, Cas9 protein was precipitated by anti-HA tag antibody, RNA components bound to Cas9 protein were eluted, and the amount of sgRNA in the elution was measured by qRT-PCR (orange bars). sgRNA amounts relative to no cellular RNA samples are represented as means ± SD (n = 3, technical triplicate).

See also Figure S1.

the amount of sgRNA binding to Cas9 by over 60% (Figure 1E). Taken together, these results supported our hypothesis that when Cas9 protein encounters cellular RNA prior to sgRNA, cellular RNA inhibits sgRNA binding to Cas9 for optimal cleavage activity. Our findings also indicate that direct transfection of Cas9 RNP

precomplexed with sgRNA is a preferable method to avoid inhibition by cellular RNA.

Improving RNP-Mediated ssODN Knockin Efficiency in iPSCs

Next, because iPSCs have more utility than HEK293T cells for disease modeling and regenerative medicine applications, we sought to optimize the RNP-mediated genome editing strategy in iPSCs in combination with ssODN donor templates to induce precise single-nucleotide editing. Previously, the ssODN knockin efficiency of several transfection methods, including Lipofectamine CRISPRMAX, Neon, and 4D-Nucleofector electroporation, has been reported (Li et al., 2016; Okamoto et al., 2019; Ran et al., 2013; Takayama et al., 2017). However, as there are limited reports using the MaxCyte electroporation platform for iPSC engineering, we optimized transfection conditions for CRISPR-Cas9 genome editing and ssODN knockin efficiency in human iPSCs.

We examined the electroporation efficiency of GFP mRNA in healthy donor-derived 1383D2 iPSCs transfected at various electroporation energy settings (E3, E6, and E8) using MaxCyte. One day after electroporation, fluorescence microscope imaging of electroporated iPSCs showed that nearly all of the cells were GFP positive, with higher GFP intensity corresponding with higher electroporation energy (Figure S2A). In line with these results, flow cytometry analysis confirmed that almost 100% of the cells were GFP positive and had an increasing GFP mean fluorescence intensity with increasing electroporation energy (Figure S2B). These results indicated that the MaxCyte electroporator can effectively transfect GFP mRNA into iPSCs. Next, we examined the indel percentage of 1383D2 iPSCs after electroporation of the Cas9/sgRNA complex targeting the human *DMD* (dystrophin) gene locus using the MaxCyte electroporator. T7E1 analysis revealed that increasing the electroporation energy also increased the percentage of indels up to 36% (Figure S2C).

Next, we selected MaxCyte condition E8 to examine the efficiency of ssODN-mediated knockin into Duchenne muscular dystrophy patient iPSCs (CiRA00111) with exon 44 deletion in the *DMD* gene. To evaluate HDR frequency, we electroporated various amounts of ssODN (DMD1) together with CRISPR RNP to disrupt the splicing acceptor and induce exon 45 skipping in the *DMD* gene. The ssODN knockin efficiency was checked by using the restriction enzyme AgeI, which is present only if ssODN-mediated HDR occurs (Figure S3A). As shown in Figure S3B, ssODN knockin was observed with as little as 1.5 μg ssODN and plateaued from 5 μg with over 60% knockin efficiency, whereas NHEJ indel efficiency was maintained regardless of ssODN amount (Figure S3C).

Optimization of ssODN-Mediated Knockin to Alter Two Nucleotides in iPSCs

We next examined the ssODN-mediated knockin efficiency by targeting the *ILF3* gene, also known as *NF110* or *NFAR2*. The *ILF3* gene encodes a dsRNA-binding protein involved in cellular host defense and is known to be phosphorylated by dsRNA-dependent protein kinase (Harashima et al., 2010). To introduce an amino acid substitution at S691C or G694A, we designed two 100-mer ssODNs to be targeted with the same sgRNA (NF110-Ex17-SA2). To assess the successful induction of the point mutations, we incorporated an additional silent mutation to introduce a blocking mutation and to generate a restriction enzyme site (BstUI or PstI), respectively (Figure S3D). With 13.3 μg ssODN, we found that MaxCyte gave better knockin efficiency than 4D-Nucleofector in iPSCs (1383D2) for both S691C (Figure S3E) and G694A (Figure S3F) substitution experiments.

One-Step Homozygous Correction of the *DYSF* Gene in Miyoshi Myopathy Patient iPSCs

To demonstrate efficient and precise genome editing, we compared the editing of a disease-associated single-nucleotide mutation with the 4D-Nucleofector and MaxCyte instruments side by side. In this experiment, we utilized the iPSC line CiRA00396 derived from a patient with Miyoshi myopathy (a form of dysferlinopathy), who has a homozygous recessive nonsense mutation of c.C3166T (p.Arg1056Ter) at exon 29 in the *Dysferlin* (*DYSF*) gene (Liu et al., 1998). Miyoshi myopathy patient iPSCs were electroporated with 6.7 or 13.3 μg ssODN, together with the Cas9/sgRNA complex. After 3 to 7 days, the iPSCs were harvested and analyzed for knockin efficiency by Hpy99I restriction enzyme digestion, which was introduced into the sgRNA targeted site upon successful ssODN knockin (Figure 2A). Although 4D-Nucleofector-mediated electroporation showed a high knockin efficiency of 46% with 6.7 μg ssODN, higher amounts of ssODN resulted in overall lower knockin efficiency. On the other hand, the MaxCyte-mediated electroporation resulted in 67% and 73% knockin efficiency at 6.7 and 13.3 μg ssODN, respectively (Figure 2B).

To obtain single edited clones, we subcloned iPSCs electroporated with CRISPR RNP and 13.3 μg ssODN using the MaxCyte instrument. Single cells were sorted and expanded, and 45 clones were examined for ssODN knockin efficiency by Sanger sequencing. Astonishingly, 73.3% (n = 33) of the clones were homozygous for the ssODN knockin, while 20% (n = 9) were heterozygous knockin and 6.7% (n = 3) showed no evidence of ssODN knockin (Figure 2C). For the heterozygous clones (n = 9) with one corrected allele by the ssODN knockin, eight also contained indels on the other allele, and the last clone contained an unedited allele. Among the three clones without ssODN knockin, two contained indels in both

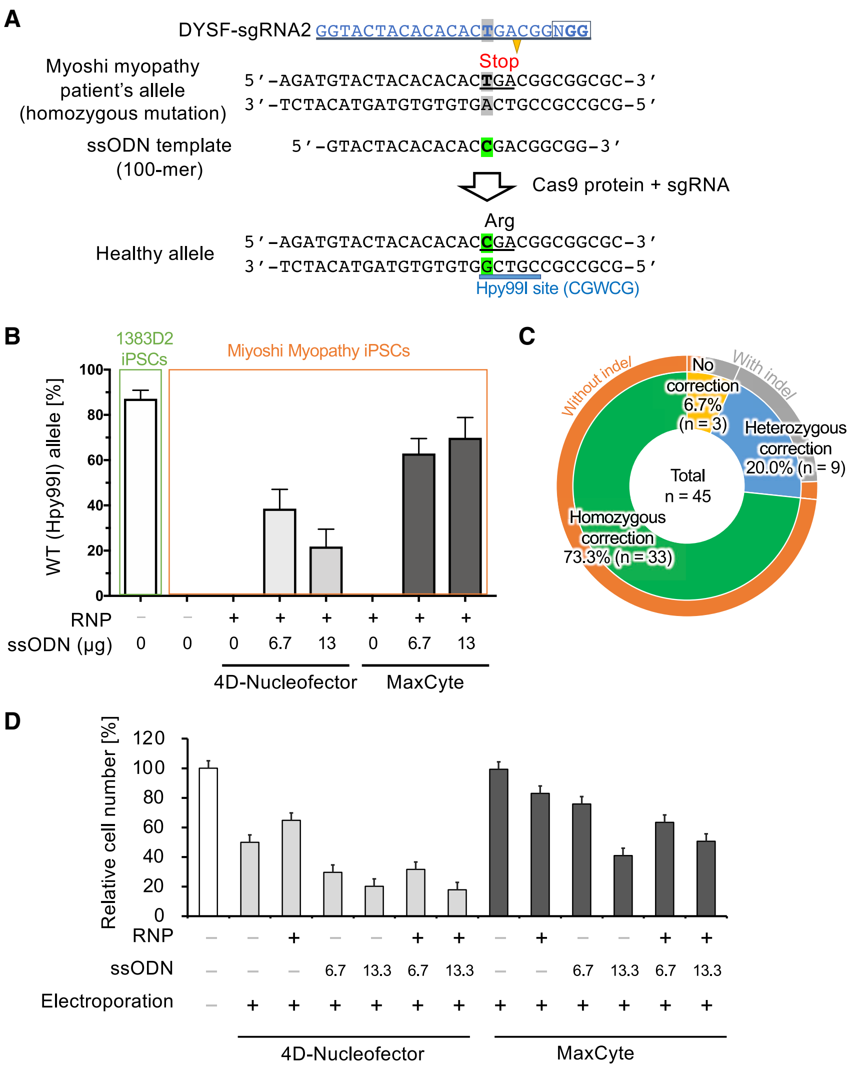

Figure 2. Correction of a Homozygous Mutation in Miyoshi Myopathy Patient iPSCs

(A) A patient with Miyoshi myopathy (dysferlinopathy) has a homozygous nonsense mutation (c.C3166T, p.Arg1056Ter, in isoform 1) at exon 29 of the *DYSF* gene on chromosome 2, which results in the loss of dysferlin protein expression. Through ssODN-mediated knockin genome editing, we attempted to correct the nonsense mutation. Successful correction can be detected by the appearance of the Hpy99I restriction enzyme site (CGWCG, W = A or T).

(B) Quantitative result of restriction enzyme digestion with Hpy99I enzyme to assess the bulk knockin efficiency in Miyoshi myopathy iPSCs (CiRA00396 clone). Digested PCR samples were analyzed by TapeStation. A PCR product from healthy 1383D2 iPSCs was used as a positive control for Hpy99I cleavage. Cleavage percentages are represented as means ± SD (n = 3, technical triplicate).

(C) After subclone isolation from MaxCyte electroporation of the ssODN template, we genotyped each subclone of Miyoshi myopathy iPSCs by Sanger sequencing. The pie chart shows the proportion of homozygously corrected subclones (green) either with or without indels. The sequences of 45 iPSC clones in total were analyzed.

(D) Cell viability assay was performed 1 day after electroporation with 4D-Nucleofector or MaxCyte by using Cell Counting Kit-8. Cell viability values relative to non-transfected sample are represented as means ± SD (n = 3, technical triplicate).

See also Figures S2–S4.

alleles and the other was unedited in both alleles. Because CRISPR-Cas9 genomic DNA cleavage may induce large deletions at the target site (Mou et al., 2017; Weisheit et al., 2020), we performed additional PCR analysis of four randomly selected homozygous knockin clones (nos. 2, 10, 12, 32) and one heterozygous knockin clone (no. 6) (Figure S4A). As shown in Figure S4B, we observed no large deletions around the targeted *DYSF* gene site of the analyzed clones. In addition, to check whether the MaxCyte genome-edited iPSCs maintained pluripotency, we checked the expression of the pluripotent markers SSEA-4 and TRA-1-60 by flow cytometry. All five subclones maintained expression of these markers comparable to the parental iPSC clone (Figure S4C). These results confirmed that RNP- and ssODN-mediated knockin using MaxCyte electroporation enabled us to obtain homozygously edited clones at very high efficiency. To explain why ssODN knockin efficiency with MaxCyte was higher than with 4D-Nucleofector, we investigated the relative viable cell number 1 day after electroporation. The results showed that transfection using MaxCyte had better survival compared with 4D-Nucleofector, especially after introduction of the ssODN (Figure 2D).

Exon Skipping in Duchenne Muscular Dystrophy Patient iPSCs

We also analyzed ssODN knockin efficiency by targeting the exon 45 splicing acceptor site of the *DMD* gene in CiRA00111 iPSCs (Figure 3A), which do not express DMD protein due to a deletion of exon 44 and can be reframed by exon 45 skipping (Gee et al., 2020; Li et al., 2015; Ousterout et al., 2015). We transfected precomplexed Cas9/sgRNA RNP with 6.7 μg ssODN by using 4D-Nucleofector and MaxCyte. After 3 to 7 days, confluent iPSCs were

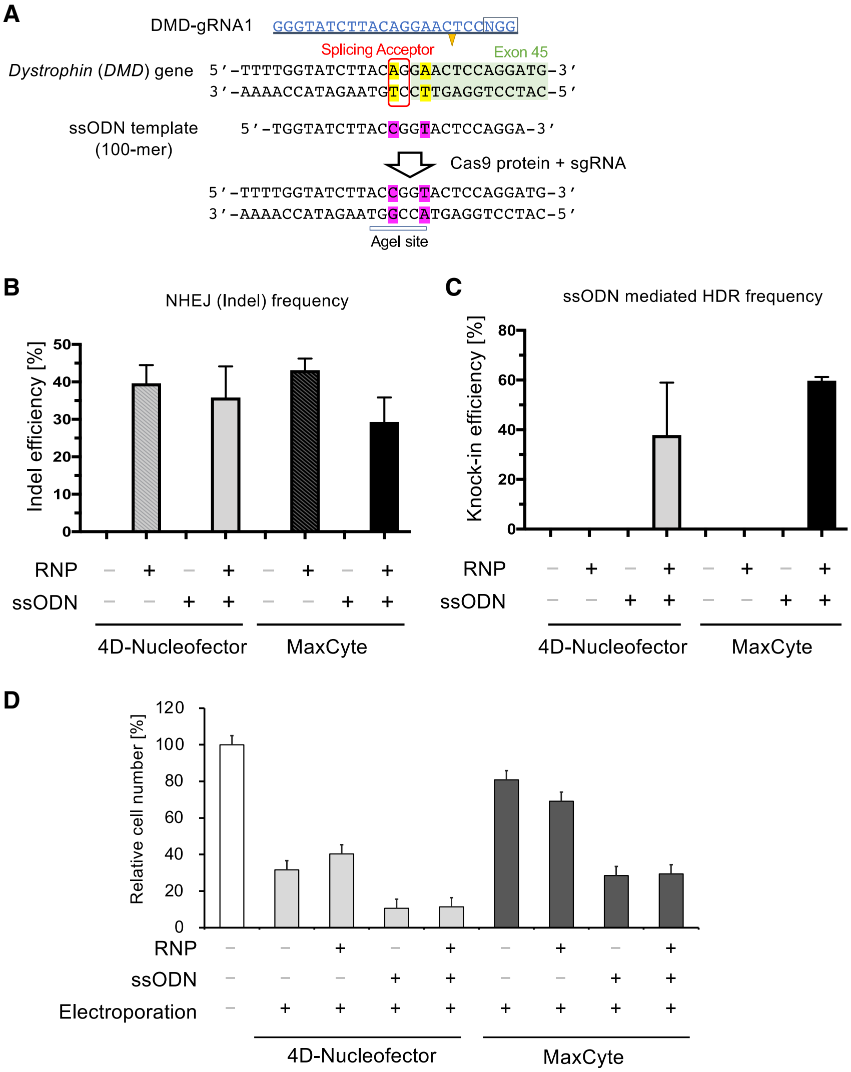

Figure 3. Disruption of a Splicing Acceptor on the *DMD* Gene

(A) To induce therapeutic exon skipping by disrupting a splicing acceptor site on exon 45 of the *DMD* gene, we designed an sgRNA and ssODN template to introduce two nucleotide alterations. Successful knockin can be evaluated by the appearance of the AgeI restriction enzyme site (A|CCGGT).

(B) We electroporated precomplexed Cas9/sgRNA together with ssODN to edit the *DMD* gene locus in DMD patient iPSCs by using either 4D-Nucleofector or MaxCyte. Three to five days after electroporation, we evaluated the NHEJ-mediated indel frequency by T7EI assay. Data are represented as means ± SD (n = 3, technical triplicate).

(C) From the same genomic DNA sample in (B), we assessed ssODN-mediated HDR efficiency by Sanger sequencing and Sequencher software. Data are represented as means ± SD (n = 3, technical triplicate).

(D) A cell counting assay was performed 1 day after electroporation by 4D-Nucleofector or MaxCyte by using Cell Counting Kit-8. Relative cell viability values are represented as means ± SD (n = 3, technical triplicate).

harvested, NHEJ-mediated indel efficiency was evaluated by T7E1 assay, and HDR-mediated precise base alteration was assessed by restriction fragment-length polymorphism analysis. T7E1 assay results showed that NHEJ-mediated cleavage activity was comparable between 4D-Nucleofector and MaxCyte (Figure 3B); however, MaxCyte showed more consistent HDR-mediated knockin efficiency (Figure 3C). When we measured cell survival 1 day after electroporation, results similar to those in Figure 2D were observed by targeting the *DMD* gene locus (Figure 3D). The addition of ssODN caused high cell toxicity, but MaxCyte electroporation attenuated this cytotoxicity in iPSCs.

High Efficiency of a *loxP* Insertion in iPSCs without Antibiotic Selection

The Cre-*loxP* system has been widely utilized for custom deletion or targeted integration in the genetics field, especially for mouse genome manipulation (Miura et al., 2018). However, insertion of a *loxP* sequence in human iPSCs has been challenging because of very low efficiency; hence, antibiotic selection has been necessary (Chen et al., 2015; Yang et al., 2017). To elucidate whether *loxP* knockin by an ssODN template without antibiotic selection is possible in iPSCs, we sequentially introduced two *loxP* sites into the dystrophin gene on chromosome X to generate a floxed allele (Figure 4A). First, we transfected RNP with the 134 bp ssODN, which contains a 34 bp *loxP* site and 50 bp homology arms on each side, to target the exon 45 region in male 1383D2 iPSCs using MaxCyte. The first ssODN-mediated *loxP* knockin was checked by PCR and restriction digestion with XmnI (Figure S5A). We could obtain >20% *loxP* insertion efficiency (Figure S5B). As above, we performed subcloning by single-cell sorting and examined the ssODN knockin efficiency by Sanger

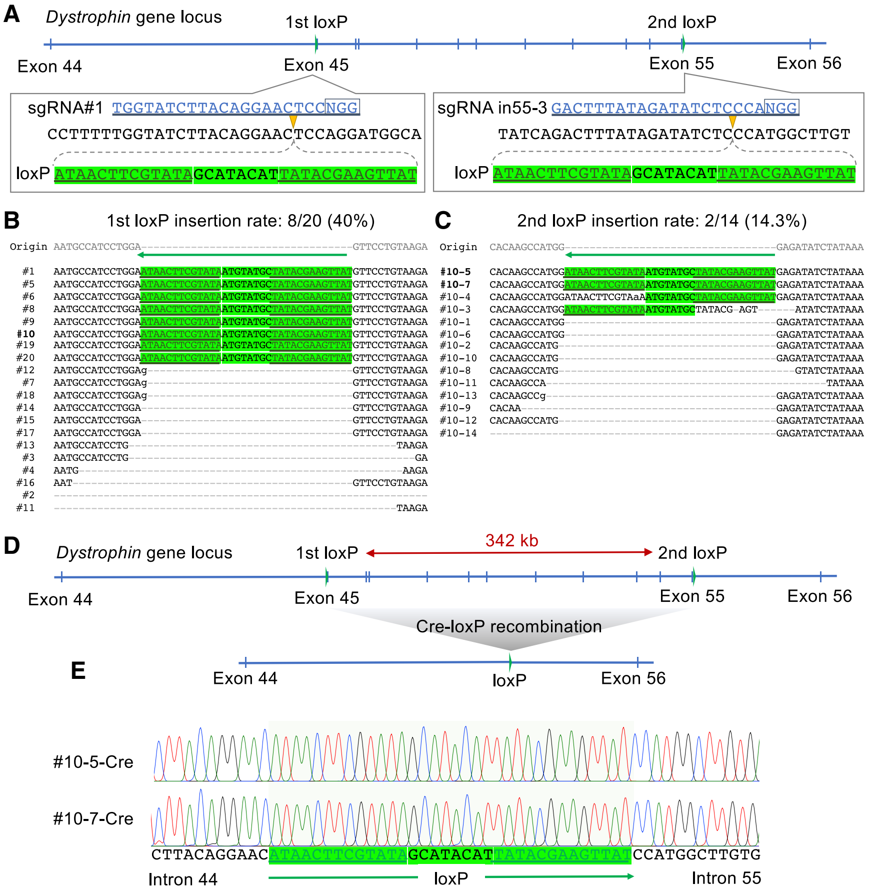

Figure 4. Insertion of Two *loxP* Sequences to Induce a Large Deletion in iPSCs

(A) Schematic of the *loxP* insertion sites in the dystrophin gene on chromosome X. Target sequence of sgRNAs and *loxP* insertion sites are indicated.

(B) After MaxCyte electroporation of Cas9 RNP, sgRNA#1, and ssODN (DMD1+*loxP*) into male iPSCs (1383D2), 20 subclones were isolated and genotyped by Sanger sequence using a reverse primer. The intact *loxP* sequence is indicated by the green marker and Cre-binding sequences in the *loxP* site are indicated by underlining.

(C) A second *loxP* site was inserted into clone 10 from (B) by MaxCyte electroporation of Cas9 RNP, sgRNA in55-3, and ssODN (DMD-in55-g3+*loxP*). Of the 14 clones analyzed by Sanger sequencing, two (nos. 10-5, 10-7) showed successful insertion of the second *loxP* site.

(D) Schematic of the Cre recombination to remove a 342 kb region on the dystrophin gene flanked by the two *loxP* sites.

(E) After transfection of the tamoxifen-inducible Cre expression plasmid vector into clone 10-5 or 10-7, genomic PCR was performed to amplify the junction of the two *loxP* sites. Sanger sequencing of the PCR product confirmed successful deletion of the 342 kb region between exon 44 and exon 55 in the dystrophin gene.
See also Figure S5.

sequencing. Of the 20 clones we analyzed, eight (40%) showed insertion of the intact *loxP* sequence at the intended site, eight (40%) were accompanied by indels or NHEJ only, and four (20%) were unedited (Figures 4B and S5C). We selected one subclone (no. 10) into which the first *loxP* was inserted (Figure S5D) and inserted a second *loxP* site after exon 55 of the dystrophin gene (342 kb distal to the first *loxP*). Of the 14 clones analyzed, two (2/14 = 14.3%) contained the intact *loxP* site successfully inserted (Figures 4C and S5E). We selected the two clones (nos. 10-5 and 10-7) containing both *loxP* sites and transfected them with a Cre expression plasmid to excise the 342 kb region (Figure 4D). Bulk PCR was performed to amplify the junction region, and it was analyzed by Sanger sequencing to confirm precise excision between the two *loxP* sites (Figure 4E). Of the total 11 clones analyzed, four showed removal of the 342 kb region flanked by the two *loxP* sites (Figure S5F), indicating the function of the two *loxP* sites. These results demonstrate that our electroporation method of precomplexed RNP and ssODN could be applied to introduce a 34 bp *loxP* site without the need for antibiotic selection.

ssODN-Mediated One-Step Biallelic Modification to Convert HLA-C1 to HLA-C2

To facilitate HLA matching for allogeneic transplantation of iPSC derivatives, the use of HLA homozygous iPSC stocks has been investigated (Sugita et al., 2020). However, HLA homozygous cells may be susceptible to attack by HLA-C1/C2 heterozygous recipient NK cells due to KIR mismatching (Ichise et al., 2017), since NK cells express KIR 2DL2/2DL3 and KIR 2DL1 receptors, which suppress HLA-C1 and HLA-C2 groups, respectively (Figure 5A). The difference between HLA-C1 (i.e., HLA-Cw1, Cw7, Cw8, Cw9) and HLA-C2 (i.e., HLA-Cw2, Cw4, Cw5, Cw6) is determined by two amino acids at the 77th and 80th positions of HLA-C protein; the HLA-C1 group has serine at position 77 and asparagine at position 80, while the HLA-C2 group has asparagine and lysine instead (Moesta et al., 2008). To avoid the HLA-C/KIR mismatch, we sought to generate HLA-C1/C2-heterozygous HLA-C alleles from HLA-C1/C1 homozygous iPSCs (Figure 5A).

To accomplish this, we chose a clinical-grade HLA homozygous iPSC line, Ff-XT28s05, which has homozygous HLA-C1 alleles (HLA-Cw7). We first designed two ssODNs;

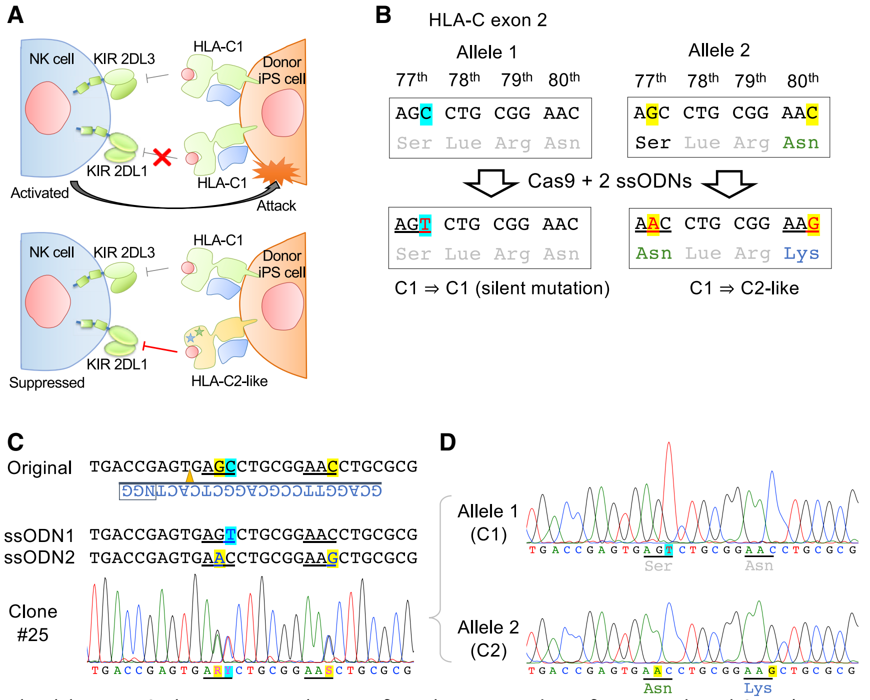

Figure 5. Simultaneous Biallelic Replacement to Generate an HLA-C1/C2 Haplotype in HLA-C1/C1 Homozygous iPSCs

(A) NK cells in HLA-C1/C2 heterozygous donors express KIR 2DL3 and KIR 2DL1 receptors, which are suppressor receptors of HLA-C1 and HLA-C2 groups, respectively. However, when cells from HLA-C1/C1 homozygous iPSCs are transplanted, these NK cells pose a risk of attacking the transplanted iPSC derivatives because of the absent inhibitory signal from the HLA-C2/KIR 2DL1 axis. Therefore, we replaced one allele of HLA-C1 with a C2-like allele to suppress KIR 2DL1.

(B) HLA-C1 and C2 groups differ by two amino acids at positions 77 and 80. We designed two ssODNs, one containing two nucleotide mutations to enable conversion from HLA-C1 to C2, and the other containing a silent mutation to prevent Cas9 from recutting the HLA-C1 allele.

(C) Ff-XT28s05 iPSCs were established from a healthy HLA-C1 homozygous donor. After electroporation of precomplexed Cas9/sgRNA with the two ssODNs by MaxCyte, 29 subclones were analyzed, and one (no. 25) showed biallelic knockin patterns by Sanger sequencing (R = A or G, Y = C or T, S = C or G).

(D) To sequence individual alleles separately, we performed TA cloning of the PCR amplicon from clone 25 and analyzed by Sanger sequencing.

one, ssODN2, contains two mutations to enable changing C1 to C2, while the other, ssODN1, contains a silent mutation to prevent Cas9 from recutting the targeted HLA-C1 locus (Figure 5B). Cas9 and sgRNA with two ssODNs were electroporated, and the knockin efficiency of each allele was 24% and 28%, respectively. Of the 29 subclones we analyzed, we could establish a clone (no. 25) that had all three intended mutations in both alleles, which we confirmed by bulk Sanger sequencing (Figure 5C) and TA cloning (Figure 5D).

DISCUSSION

While investigating various delivery methods (plasmid DNA vector, RNP, and Dox-inducible *piggyBac* vector) of the CRIPSR-Cas9/sgRNA system, we observed that exogenous plasmid DNA and an integrated *piggyBac* vector could mediate high levels of Cas9 protein expression in cells, but genome editing efficiency was comparable to or even lower than RNP transfection, which resulted in lower amounts of Cas9 protein in cells. To investigate the underlying mechanistic reasons behind this phenomenon, we found that free Cas9 protein (or apo-Cas9) could be inhibited by cellular RNA for binding with sgRNAs *in vitro*, which dramatically reduced Cas9's DNA cleavage efficiency. This effect suggests that Cas9 is susceptible to inhibition by intracellular RNAs when Cas9 protein is expressed. On the other hand, Cas9 protein precomplexed with sgRNA *in vitro* is less susceptible to inhibition by intracellular RNAs. Hence, precomplexed RNP transfection is preferable for achieving high genome editing efficiency compared with expression-based delivery systems.

Recently, several anti-CRISPR proteins (AcrIIs) have been identified in bacteriophages that specifically inhibit type II CRISPR-Cas9 activities (Fuchsbauer et al., 2019; Harrington et al., 2017; Jiang et al., 2019). For instance, AcrIIA1-AcrIIA6 binds to Cas9 and inhibits DNA binding in an allosteric manner. AcrIIC3 also binds to *Neisseria meningitidis* Cas9 (NmCas9) for blocking the DNA loading step (Zhu et al., 2019). Other anti-CRISPR proteins, such as AcrIIA11 and AcrIIC1, prevent DNA cleavage. To date, only AcrIIC2 has been identified as an anti-CRISPR to prevent sgRNA complexing with NmCas9 (Zhu et al., 2019). Although it is unclear how intracellular RNA inhibits Cas9 for sgRNA loading at the molecular level, we speculate that cellular RNA binds to cationic Cas9 protein (pI 9.01) non-specifically and causes steric hindrance or physically obstructs sgRNA from getting into close proximity to apo-Cas9 (Lim et al., 2016).

In our experiments, up to 4,000 ng RNA was used per 10–20 μL reaction volume; hence, the maximum RNA concentration was 0.4 mg/μL. On the other hand, the cellular RNA concentration is roughly 10–30 mg/μL based on estimating

the amount of total cellular RNA per human cell (10–30 pg, BNID 111205) and the cellular volume (4×10^{-6} μL, BNID 105906). Therefore, the physiological concentration of intracellular RNA is one or two orders greater than the concentration range used in our experiments.

Other possible implications of our findings are the importance of Cas9 protein purification when extracted from a crude lysate (Qiao et al., 2019), as contaminated bacterial RNA may potentially compromise Cas9 genome editing activity or lead to a lot-to-lot variation in enzymatic activity. Notably, as shown in Figure 1D, the inhibition of sgRNA loading can be released by RNase treatment. Recently, RNP electroporation has become a standard genome editing method, owing to its simplicity and high efficiency. Here, we provide a plausible explanation for why precomplexed RNP offers higher genome editing specificity compared with other expression methods.

To further optimize RNP-mediated genome editing, we compared electroporation instruments. Although the Lonza 4D-Nucleofector has been widely used (Skarnes et al., 2019), we demonstrated that the MaxCyte electroporation platform is also a suitable instrument for genome editing applications. When we compared the 4D-Nucleofector and MaxCyte instruments side by side, we found that knockin efficiency with ssODN was higher with the MaxCyte platform (Figures 2 and 3). Moreover, MaxCyte enabled predominantly homozygous knockin efficiencies in as many as 72% of subclones at the *DYSF* gene locus in Miyoshi myopathy patient iPSCs (Figure 2).

To promote the HDR pathway over NHEJ, the availability of the donor template at the vicinity of a DSB site is critical. Transfecting a high concentration of ssODN is advantageous to increase the chance for the ssODN to reach the nucleus and be in close proximity to the Cas9 cleavage site; however, ssODN itself is toxic for cells, as shown in Figures 2D and 3D. Because the HDR DNA repair pathway mainly takes place at S or G2 phase of the cell cycle, we speculate that actively proliferating cells should have a higher HDR rate than damaged arrested cells. The different performances of the two electroporation instruments were accompanied by a difference in cell viability, suggesting this parameter could affect the HDR frequency.

Applying our optimized and robust ssODN knockin approach to engineering human iPSCs, we achieved insertion of a 34 bp *loxP* sequence into a desired locus in up to 40% of cells, which can be used as a hub for site-specific integration. Furthermore, we demonstrated that two *loxP* sites could be inserted into a target gene locus for introducing a floxed allele. Upon the introduction of Cre recombinase, a precise and large deletion of 342 kb could be introduced in iPSCs.

We also investigated the knockin of two unique ssODNs into both alleles, which enabled us to generate an HLA-C1/C2 heterozygous allele from HLA-C1/C1 homozygous iPSCs. Engineered HLA-C1/C2 iPSCs should be able to suppress NK cells from HLA-C1/C2 donors via the KIR 2DL1 receptor. Taken together, our study would facilitate custom editing of a desired genomic locus with precomplexed RNP and ssODN templates in iPSCs for genetic research and regenerative medicine applications.

EXPERIMENTAL PROCEDURES

Cell Culture

HEK293T cells (ATCC, cat. no. CRL-3216) and CRONUS-HEK293T cells were cultured in DMEM (Nacalai Tesque, cat. no. 08459-64) with 10% FBS (Biosera, cat. no. FB-1365). Feeder-free iPSCs were maintained in StemFit AK03N (Ajinomoto) in a laminin 511 E8 (iMatrix-511 E8, cat. no. 892012, Nippi)-coated six well plate (BD, cat. no. 353046). TrypLE Select (Thermo Fisher Scientific, cat. no. 12563-011) was used for passaging and harvesting cells. 1383D2 iPSCs (donor: healthy Asian male, 36 years of age at the time of donation) were established by Dr. Masato Nakagawa (Nakagawa et al., 2014) and provided by FiT (Facility for iPSC Therapy) at CiRA. DMD-iPSCs (CiRA00111 clone) from a patient with Duchenne muscular dystrophy with exon 44 deletion were described previously (Li et al., 2015). Miyoshi myopathy patient iPSCs (CiRA00396 clone) derived from a patient with a homozygous nonsense mutation (exon 29; c.C3166T, p.Arg1056Ter) were a kind gift from Dr. Hidetoshi Sakurai (CiRA). The protocol to establish the patient-derived iPSC lines was approved by the ethics committee of the Graduate School and Faculty of Medicine, Kyoto University (approval nos. R0091 and G259). Ff-XT28s05 iPSCs were generated from a healthy donor homozygous for the most frequent HLA haplotypes in Japan (A*24:02, B*07:02, C*07:02, DRB1*01:01) under a clinical-grade cell manufacturing protocol at FiT in CiRA, Kyoto University.

Electroporation of RNP by MaxCyte or 4D-Nucleofector

The day before electroporation, iPSCs were seeded onto a six well plate coated with iMatrix-511 at a density of 1.5–2×10^6 cells per well. On the day of transfection, iPSCs were washed with 2 mL PBS and then incubated with 0.5 mL Accutase or TrypLE Select for 10 min at 37°C. The cells were then detached from the plate by pipetting, transferred to a 15 mL centrifuge tube containing AK03N with 10 μM ROCK inhibitor (Y-27632, Wako, cat. no 253-00514), and centrifuged for 5 min at 120 × *g* to harvest the cells by discarding the supernatant.

For MaxCyte electroporation, the cells were resuspended in 1 mL MaxCyte buffer. The cells were counted, washed with 5 mL MaxCyte buffer, and resuspended in MaxCyte buffer at a density of 2.5×10^7 cells/mL. For RNP electroporation, 10 μg recombinant SpCas9 protein (IDT, cat. no. 1081058, or Thermo Fisher, cat. no. A36498) and 2.5 μg IVT sgRNA were incubated for 5 min at room temperature, and then 1.25×10^6 cells with 50 μL MaxCyte buffer and 0.5 to 25 μg ssODN (Fasmac, Table S3) were added and mixed gently before transfer to an OC-100 cuvette. For GFP mRNA electroporation, 5 μg mRNA (TriLink) was used. Electroporation was carried out on the MaxCyte ATX

or STX at the indicated electroporation energy. Immediately after electroporation, the OC-100 cuvettes with iPSCs were incubated at 37°C for 20 min in a humidified incubator to allow for cell membrane recovery. Finally, cells were added to a single well of an iMatrix-511-coated six well plate containing 2 mL prewarmed Stem Fit AK03N medium with 10 μM Y-27632. Semi-confluent cells (3–7 days after transfection) were harvested for genomic DNA extraction and/or FACS analysis.

For 4D-Nucleofector electroporation, the cells were resuspended in StemFit AK03N medium and cell counting was performed. After centrifugation, 3×10^5 cells were gently resuspended in 20 μL P4 Primary Cell Nucleofector Solution from a P4 Primary Cell 4D-Nucleofector Kit (Lonza, cat. no. V4XP-4032). For RNP electroporation, 5 μg recombinant SpCas9 protein (IDT, cat. no. 1081058, or Thermo Fisher, cat. no. A36498) and 1.25 μg IVT sgRNA were incubated for 5 min at room temperature and then added to the cell suspension with or without ssODN (ranging from 0.5 to 25 μg). The cells were gently mixed by pipetting five times and then transferred to a well of a 16 well Nucleocuvette Strip. Immediately after electroporation using the CA-137 protocol of 4D-Nucleofector, the cells were transferred to an iMatrix-511-coated well of a six well plate containing 2 mL prewarmed StemFit AK03N medium with 10 μM Y-27632. Semi-confluent cells (3–7 days post-transfection) were harvested for genomic DNA extraction and/or FACS analysis as described above.

loxP Insertion by Cas9/sgRNA and ssODN Electroporation

iPSCs (1383D2 clone) were genome edited as described in the MaxCyte electroporation section. For 1.0×10^6 cells with 50 μL MaxCyte buffer, 5 μg Cas9 protein (Thermo Fisher, cat. no. A36498), 1.25 μg IVT sgRNA (either DMD-sgRNA1 or DMD-in55-g3), and 6 μg ssODN with *loxP* sequence (134-mer, either "DMD1+loxP-ssODN" or "DMD-in55-g3+loxP-ssODN," see Table S4) were electroporated using MaxCyte with Optimization Energy 8. After confirmation of the *loxP* knockin efficiency in bulk-electroporated cells, single-cell clones were isolated by using a BD FACSAria cell sorter (BD Biosciences) or by limited dilution. For the Cre-*loxP* excision experiment, floxed subclones (nos. 10-5 and 10-7) were seeded into a 24-well plate (1.5–3.0 $\times 10^6$ cells/well) and transfected with 500 ng tamoxifen-inducible Cre-expressing plasmid pCAG-IP-MerCreMer (a kind gift from Dr. Makoto Tachibana, Osaka University) with 1.5 μL Lipofectamine Stem Transfection Reagent (Thermo Fisher, cat. no. STEM00015). Four to six hours after the lipofection, the culture medium was replaced with 1 μM 4-hydroxytamoxifen (Sigma-Aldrich, cat. no. H7904) to induce nuclear translocation of Cre-recombinase fused with modified estrogen receptor (Mer). After confirmation of the excision of the floxed site by genomic PCR, two subclones from clone 10-5 and nine subclones from clone 10-7 were established and genotyped by PCR with "DMDexon55(45-55)check_dir5" and "DMDexon45(45-55) check_rev5" primers (Table S1).

SUPPLEMENTAL INFORMATION

Supplemental Information can be found online at https://doi.org/10.1016/j.stemcr.2021.02.013.

AUTHOR CONTRIBUTIONS

Conceptualization, A.K., M.L., H.X., P.G., and A.H.; investigation, A.K., M.L., H.X., T.I., M.O., N.S., Y.K., X.W., and P.G.; writing – original draft, A.K., M.L., P.G., and H.X.; writing – review & editing, P.G. and A.H.; supervision, P.G. and A.H.; funding acquisition, A.H.

CONFLICTS OF INTEREST

P.G. is currently an employee of MaxCyte, Inc. All other authors declare no competing interests.

ACKNOWLEDGMENTS

We would like to thank Dr. Hidetoshi Sakurai for providing CiRA00396 iPSCs, Dr. Masato Nakagawa for providing 1383D2 iPSCs, CiRA FiT for providing Ff-XT28s05 iPSCs, and Dr. Peter Karagiannis for proofreading the manuscript. We are also grateful to Joseph A. Abad, Brad Calvin, and Jim Brady for technical support, usage of the MaxCyte instrument, and providing reagents to conduct electroporation experiments. This study is founded by Japan Agency for Medical Research and Development (AMED) grants (JP19bm0104001, JP19bm0804005, JP19ek0109293, JP19bm0404037) and a National Center of Neurology and Psychiatry (NCNP) grant (no.28-6).

Received: August 14, 2020
Revised: February 12, 2021
Accepted: February 12, 2021
Published: March 11, 2021

REFERENCES

Anzalone, A.V., Koblan, L.W., and Liu, D.R. (2020). Genome editing with CRISPR-Cas nucleases, base editors, transposases and prime editors. Nat. Biotechnol. *38*, 824–844.

Banan, M. (2020). Recent advances in CRISPR/Cas9-mediated knock-ins in mammalian cells. J. Biotechnol. *308*, 1–9.

Bertero, A., Pawlowski, M., Ortmann, D., Snijders, K., Yiangou, L., Cardoso de Brito, M., Brown, S., Bernard, W.G., Cooper, J.D., Giacomelli, E., et al. (2016). Optimized inducible shRNA and CRISPR/Cas9 platforms for in vitro studies of human development using hPSCs. Development *143*, 4405–4418.

Carlson-Stevermer, J., Abdeen, A.A., Kohlenberg, L., Goedland, M., Molugu, K., Lou, M., and Saha, K. (2017). Assembly of CRISPR ribonucleoproteins with biotinylated oligonucleotides via an RNA aptamer for precise gene editing. Nat. Commun. *8*, 1711.

Chen, Y., Cao, J., Xiong, M., Petersen, A.J., Dong, Y., Tao, Y., Huang, C.T.-L., Du, Z., and Zhang, S.-C. (2015). Engineering human stem cell lines with inducible gene knockout using CRISPR/Cas9. Cell Stem Cell *17*, 233–244.

Dastidar, S., Ardui, S., Singh, K., Majumdar, D., Nair, N., Fu, Y., Reyon, D., Samara, E., Gerli, M.F.M., Klein, A.F., et al. (2018). Efficient CRISPR/Cas9-mediated editing of trinucleotide repeat expansion in myotonic dystrophy patient-derived iPS and myogenic cells. Nucleic Acids Res. *46*, 8275–8298.

Dow, L.E., Fisher, J., O'Rourke, K.P., Muley, A., Kastenhuber, E.R., Livshits, G., Tschaharganeh, D.F., Socci, N.D., and Lowe, S.W.

(2015). Inducible in vivo genome editing with CRISPR-Cas9. Nat. Biotechnol. *33*, 390–394.

Fuchsbauer, O., Swuec, P., Zimberger, C., Amigues, B., Levesque, S., Agudelo, D., Duringer, A., Chaves-Sanjuan, A., Spinelli, S., Rousseau, G.M., et al. (2019). Cas9 allosteric inhibition by the anti-CRISPR protein AcrIIA6. Mol. Cell *76*, 922–937.e7.

Gee, P., Lung, M.S.Y., Okuzaki, Y., Sasakawa, N., Iguchi, T., Makita, Y., Hozumi, H., Miura, Y., Yang, L.F., Iwasaki, M., et al. (2020). Extracellular nanovesicles for packaging of CRISPR-Cas9 protein and sgRNA to induce therapeutic exon skipping. Nat. Commun. *11*, 1334.

Harashima, A., Guettouche, T., and Barber, G.N. (2010). Phosphorylation of the NFAR proteins by the dsRNA-dependent protein kinase PKR constitutes a novel mechanism of translational regulation and cellular defense. Genes Dev. *24*, 2640–2653.

Harrington, L.B., Doxzen, K.W., Ma, E., Liu, J.-J., Knott, G.J., Edraki, A., Garcia, B., Amrani, N., Chen, J.S., Cofsky, J.C., et al. (2017). A broad-spectrum inhibitor of CRISPR-Cas9. Cell *170*, 1224–1233.e5.

Hockemeyer, D., and Jaenisch, R. (2016). Induced pluripotent stem cells meet genome editing. Cell Stem Cell *18*, 573–586.

Hotta, A., and Yamanaka, S. (2015). From genomics to gene Therapy: induced pluripotent stem cells meet genome editing. Annu. Rev. Genet. *49*, 47–70.

Ichise, H., Nagano, S., Maeda, T., Miyazaki, M., Miyazaki, Y., Kojima, H., Yawata, N., Yawata, M., Tanaka, H., Saji, H., et al. (2017). NK cell alloreactivity against KIR-ligand-mismatched HLA-haploidentical tissue derived from HLA haplotype-homozygous iPSCs. Stem Cell Reports *9*, 853–867.

Ishida, K., Xu, H., Sasakawa, N., Lung, M.S.Y., Kudryashev, J.A., Gee, P., and Hotta, A. (2018). Site-specific randomization of the endogenous genome by a regulatable CRISPR-Cas9 piggyBac system in human cells. Sci. Rep. *8*, 310.

Jiang, F., Liu, J.-J., Osuna, B.A., Xu, M., Berry, J.D., Rauch, B.J., Nogales, E., Bondy-Denomy, J., and Doudna, J.A. (2019). Temperature-responsive competitive inhibition of CRISPR-Cas9. Mol. Cell *73*, 601–610.e5.

Jinek, M., Chylinski, K., Fonfara, I., Hauer, M., Doudna, J.A., and Charpentier, E. (2012). A programmable dual-RNA-guided DNA endonuclease in adaptive bacterial immunity. Science *337*, 816–821.

Kim, S., Kim, D., Cho, S.-W., Kim, J., and Kim, J.-S. (2014). Highly efficient RNA-guided genome editing in human cells via delivery of purified Cas9 ribonucleoproteins. Genome Res. *24*, 1012–1019.

Li, H.L., Fujimoto, N., Sasakawa, N., Shirai, S., Ohkame, T., Sakuma, T., Tanaka, M., Amano, N., Watanabe, A., Sakurai, H., et al. (2015). Precise correction of the dystrophin gene in duchenne muscular dystrophy patient induced pluripotent stem cells by TALEN and CRISPR-Cas9. Stem Cell Reports *4*, 143–154.

Li, H.L., Gee, P., Ishida, K., and Hotta, A. (2016). Efficient genomic correction methods in human iPS cells using CRISPR-Cas9 system. Methods *101*, 27–35.

Liang, X., Potter, J., Kumar, S., Zou, Y., Quintanilla, R., Sridharan, M., Carte, J., Chen, W., Roark, N., Ranganathan, S., et al. (2015). Rapid and highly efficient mammalian cell engineering via Cas9 protein transfection. J. Biotechnol. *208*, 44–53.

Lim, Y., Bak, S.Y., Sung, K., Jeong, E., Lee, S.H., Kim, J.-S., Bae, S., and Kim, S.K. (2016). Structural roles of guide RNAs in the nuclease activity of Cas9 endonuclease. Nat. Commun. *7*, 13350–13358.

Lin, S., Staahl, B.T., Alla, R.K., and Doudna, J.A. (2014). Enhanced homology-directed human genome engineering by controlled timing of CRISPR/Cas9 delivery. Elife *3*, e04766.

Liu, J., Aoki, M., Illa, I., Wu, C., Fardeau, M., Angelini, C., Serrano, C., Urtizberea, J.A., Hentati, F., Hamida, M.B., et al. (1998). Dysferlin, a novel skeletal muscle gene, is mutated in Miyoshi myopathy and limb girdle muscular dystrophy. Nat. Genet. *20*, 31–36.

Ma, X., Chen, X., Jin, Y., Ge, W., Wang, W., Kong, L., Ji, J., Guo, X., Huang, J., Feng, X.-H., et al. (2018). Small molecules promote CRISPR-Cpf1-mediated genome editing in human pluripotent stem cells. Nat. Commun. *9*, 1303–1307.

Martin, R.M., Ikeda, K., Cromer, M.K., Uchida, N., Nishimura, T., Romano, R., Tong, A.J., Lemgart, V.T., Camarena, J., Pavel-Dinu, M., et al. (2019). Highly efficient and marker-free genome editing of human pluripotent stem cells by CRISPR-Cas9 RNP and AAV6 donor-mediated homologous recombination. Cell Stem Cell *24*, 821–828.e5.

Miura, H., Quadros, R.M., Gurumurthy, C.B., and Ohtsuka, M. (2018). Easi-CRISPR for creating knock-in and conditional knockout mouse models using long ssDNA donors. Nat. Protoc. *13*, 195–215.

Miyaoka, Y., Berman, J.R., Cooper, S.B., Mayerl, S.J., Chan, A.H., Zhang, B., Karlin-Neumann, G.A., and Conklin, B.R. (2016). Systematic quantification of HDR and NHEJ reveals effects of locus, nuclease, and cell type on genome-editing. Sci. Rep. *6*, 23549.

Miyaoka, Y., Chan, A.H., Judge, L.M., Yoo, J., Huang, M., Nguyen, T.D., Lizarraga, P.P., So, P.-L., and Conklin, B.R. (2014). Isolation of single-base genome-edited human iPS cells without antibiotic selection. Nat. Methods *11*, 291–293.

Moesta, A.K., Norman, P.J., Yawata, M., Yawata, N., Gleimer, M., and Parham, P. (2008). Synergistic polymorphism at two positions distal to the ligand-binding site makes KIR2DL2 a stronger receptor for HLA-C than KIR2DL3. J. Immunol. *180*, 3969–3979.

Mou, H., Smith, J.L., Peng, L., Yin, H., Moore, J., Zhang, X.-O., Song, C.-Q., Sheel, A., Wu, Q., Ozata, D.M., et al. (2017). CRISPR/Cas9-mediated genome editing induces exon skipping by alternative splicing or exon deletion. Genome Biol. *18*, 108.

Nakagawa, M., Taniguchi, Y., Senda, S., Takizawa, N., Ichisaka, T., Asano, K., Morizane, A., Doi, D., Takahashi, J., Nishizawa, M., et al. (2014). A novel efficient feeder-free culture system for the derivation of human induced pluripotent stem cells. Sci. Rep. *4*, 3594.

Okamoto, S., Amaishi, Y., Maki, I., Enoki, T., and Mineno, J. (2019). Highly efficient genome editing for single-base substitutions using optimized ssODNs with Cas9-RNPs. Sci. Rep. *9*, 4811.

Ousterout, D.G., Kabadi, A.M., Thakore, P.I., Majoros, W.H., Reddy, T.E., and Gersbach, C.A. (2015). Multiplex CRISPR/Cas9-based genome editing for correction of dystrophin mutations that cause Duchenne muscular dystrophy. Nat. Commun. *6*, 6244.

Qiao, J., Li, W., Lin, S., Sun, W., Ma, L., and Liu, Y. (2019). Co-expression of Cas9 and single-guided RNAs in Escherichia coli streamlines production of Cas9 ribonucleoproteins. Commun. Biol. *2*, 161–166.

Ran, F.A., Hsu, P.D., Wright, J., Agarwala, V., Scott, D.A., and Zhang, F. (2013). Genome engineering using the CRISPR-Cas9 system. Nat. Protoc. *8*, 2281–2308.

Rees, H.A., and Liu, D.R. (2018). Base editing: precision chemistry on the genome and transcriptome of living cells. Nat. Rev. Genet. *19*, 770–788.

Riesenberg, S., and Maricic, T. (2018). Targeting repair pathways with small molecules increases precise genome editing in pluripotent stem cells. Nat. Commun. *9*, 2164–2169.

Savic, N., Ringnalda, F.C., Lindsay, H., Berk, C., Bargsten, K., Li, Y., Neri, D., Robinson, M.D., Ciaudo, C., Hall, J., et al. (2018). Covalent linkage of the DNA repair template to the CRISPR-Cas9 nuclease enhances homology-directed repair. Elife *7*, e33761.

Skarnes, W.C., Pellegrino, E., and McDonough, J.A. (2019). Improving homology-directed repair efficiency in human stem cells. Methods *164-165*, 18–28.

Sugita, S., Mandai, M., Hirami, Y., Takagi, S., Maeda, T., Fujihara, M., Matsuzaki, M., Yamamoto, M., Iseki, K., Hayashi, N., et al. (2020). HLA-matched allogeneic iPS cells-derived RPE transplantation for macular degeneration. J. Clin. Med. *9*, 2217.

Takahashi, K., and Yamanaka, S. (2016). A decade of transcription factor-mediated reprogramming to pluripotency. Nat. Rev. Mol. Cell Biol. *17*, 183–193.

Takahashi, K., Tanabe, K., Ohnuki, M., Narita, M., Ichisaka, T., Tomoda, K., and Yamanaka, S. (2007). Induction of pluripotent stem cells from adult human fibroblasts by defined factors. Cell *131*, 861–872.

Takayama, K., Igai, K., Hagihara, Y., Hashimoto, R., Hanawa, M., Sakuma, T., Tachibana, M., Sakurai, F., Yamamoto, T., and Mizuguchi, H. (2017). Highly efficient biallelic genome editing of human ES/iPS cells using a CRISPR/Cas9 or TALEN system. Nucleic Acids Res. *45*, 5198–5207.

Weisheit, I., Kroeger, J.A., Malik, R., Klimmt, J., Crusius, D., Dannert, A., Dichgans, M., and Paquet, D. (2020). Detection of deleterious on-target effects after HDR-mediated CRISPR editing. Cell Rep. *31*, 107689.

Xu, H., Wang, B., Ono, M., Kagita, A., Fujii, K., Sasakawa, N., Ueda, T., Gee, P., Nishikawa, M., Nomura, M., et al. (2019). Targeted disruption of HLA genes via CRISPR-Cas9 generates iPSCs with enhanced immune compatibility. Cell Stem Cell *24*, 566–578.e7.

Yang, D., Scavuzzo, M.A., Chmielowiec, J., Sharp, R., Bajic, A., and Borowiak, M. (2016). Enrichment of G2/M cell cycle phase in human pluripotent stem cells enhances HDR-mediated gene repair with customizable endonucleases. Sci. Rep. *6*, 861.

Yang, F., Liu, C., Chen, D., Tu, M., Xie, H., Sun, H., Ge, X., Tang, L., Li, J., Zheng, J., et al. (2017). CRISPR/Cas9-loxP-Mediated gene editing as a novel site-specific genetic manipulation tool. Mol. Ther. Nucleic Acids *7*, 378–386.

Yu, C., Liu, Y., Ma, T., Liu, K., Xu, S., Zhang, Y., Liu, H., La Russa, M., Xie, M., Ding, S., et al. (2015). Small molecules enhance CRISPR genome editing in pluripotent stem cells. Cell Stem Cell *16*, 142–147.

Zhu, Y., Gao, A., Zhan, Q., Wang, Y., Feng, H., Liu, S., Gao, G., Serganov, A., and Gao, P. (2019). Diverse mechanisms of CRISPR-Cas9 inhibition by type IIC anti-CRISPR proteins. Mol. Cell *74*, 296–309.e7.

Stem Cell Reports, Volume *16*

Supplemental Information

Efficient ssODN-Mediated Targeting by Avoiding Cellular Inhibitory RNAs through Precomplexed CRISPR-Cas9/sgRNA Ribonucleoprotein

Akihiro Kagita, Mandy S.Y. Lung, Huaigeng Xu, Yuto Kita, Noriko Sasakawa, Takahiro Iguchi, Miyuki Ono, Xiou H. Wang, Peter Gee, and Akitsu Hotta

Experimental Procedures

In vitro cleavage assay to measure Cas9's nuclease activity

For one sample, 50 ng recombinant SpCas9 (PNA Bio, Cat. No. CP01; IDT, Cat. No. 1081058; or Thermo Fisher, Cat. No. A36498) was mixed by gentle pipetting with 12.5 ng in vitro transcribed DMD1 sgRNA and 10 ng of a 703 bp PCR amplicon from the human *DMD* gene locus in a total volume of 10 to 20 µL cleavage buffer containing a final concentration of 20 mM HEPES (pH 7.5), 150 mM KCl, 1 mM DTT, 1 mM MgCl2, 50 µg/mL BSA and 10% glycerol and incubated at 37°C for 1 hour. The samples were then treated with 40 ng RNase A at 37°C for 30 minutes and 10 µg Proteinase K at 50°C for 20 minutes to remove residual RNA and protein before the visualization of cleaved DNA bands using the Agilent 2200 TapeStation system.

Intracellular Cas9 activity and quantification measurements by various transfection methods

For plasmid DNA transfection, HEK293T cells were seeded into 6-well plates and transfected with plasmid DNA vectors pHL-EF1a-hcSpCas9-A (2 µg, Addgene #60599) and pHL-H1-sgRNA-EF1a-RiH (2 µg, Addgene #60601) by Lipofectamine 2000 (20 µL). 48 hours after the transfection, bulk cells were harvested for Western blot and T7EI analyses. For Cas9 protein and gRNA transfection, HEK293T cells were seeded into 6-well plates and transfected with either Cas9 protein pre-mixed with sgRNA (RNP pre-mix) or Cas9 protein/sgRNA separately (RNP post-mix) by using Lipofectamine CRISPRMAX. The bulk cells were harvested 4 hours after transfection for Western blot analysis and at 48 hours after the transfection for T7EI analysis. For CRONUS piggyBac vector with Dox/Dex induction, previously established CRONUS-HEK293T cells (Ishida et al., 2018) were seeded into a 6-well plate with (CRONUS[+]) or without (CRONUS[−]) doxycycline (Dox, 2 µM) and dexamethasone (Dex, 1 µM). After 48 hours of the Dox and Dex treatment, the bulk cells were harvested for western blot and T7EI analyses. Note, the leaky expression of CRONUS-Cas9 might be due to the use of FBS, which was not tetracycline-free.

Western blotting

Whole cell pellets were lysed with Lysis buffer (20 mM Tris-HCl, pH=7.5, 150 mM NaCl, 1 mM EDTA, 1% Triton-X) and supplemented with cOmplete protease inhibitor cocktail (Merck, 11873580001) for 15 min and centrifuged (14 000 rpm for 10 min at 4ºC) to remove the insoluble fraction. Total protein concentration was measured by the Bradford assay (BioRad, Cat No. 5000001JA) using a BSA standard curve. After mixing with Sample buffer with 200 mM DTT and denatured with 98ºC for 5 min, we loaded 0.2-0.5 µg total protein per lane on 4-12% gradient gel (GeneScript) for SDS-PAGE in MOPS buffer. After electrophoresis (150 V, 80 mA, 60 min), the gel was equilibrated with 10% methanol in 1× blotting buffer for 10 min. The protein samples were blotted onto a nitrocellulose membrane by using the iBlot Dry Blotting system with P0 program (ThermoFisher). After washing the blotted nitrocellulose membrane with PBS-T for 5 min, the blot was incubated with Blocking One blocking solution (Nacalai tesque) for 1 hour at room temperature.

Streptococcus pyogenes Cas9 protein (160 kDa) was detected with anti-Cas9 mouse monoclonal antibody (Active motif, clone 7A9-3A3, 1:1000 dilution) in 2 mL Can Get Signal 1 solution (Toyobo, NKB-101) and incubated overnight at 4ºC. After washing by PBS-T 3 times, bands were detected with HRP linked anti-mouse IgG secondary antibody (Cell Signaling Technology, #7076, 1:1000 dilution) in 2 mL Can Get Signal 2 solution (Toyobo) for 1 hour at room temperature and imaged with Amersham ECL Western Blot Detection Reagent (GE Healthcare) by using the ChemiDoc XRS+ Imaging system (BioRad). Then, the same membrane was washed with PBS-T two times and stripped with Restore PLUS Western Blot Stripping buffer (ThermFisher, Cat No. 46430) for 30 min at 37ºC. After washing with PBS-T 2-3 times, the membrane was incubated with BlockOne blocking solution for 1 hour at room temperature. As a cell lysate loading control, ACTB (β-actin, 42 kDa) protein was detected with anti-ACTB mouse monoclonal antibody (Sigma-Aldrich, clone AC-15, 1:2000 dilution) in 2 mL Can Get Signal 1 solution and incubated overnight at 4ºC. After washing by PBS-T 3 times, bands were detected with HRP-linked anti-mouse IgG secondly antibody (1:1000 dilution) in 2 mL Can Get Signal 2 solution for 1 hour at room temperature and imaged with Amersham ECL Western Blot Detection Reagent by using the ChemiDoc XRS+ Imaging system.

T7E1 assay

Extracted genomic DNA (100 ng) was used as a template for PCR amplification of the genomic region of interest using PrimeSTAR GXL DNA polymerase (Toyobo, Cat. No. R050A) and primers in **Supplemental Table 1** The resulting PCR amplicon was column purified with a Wizard SV Gel and PCR Clean-Up System (Promega, Cat. No. A9285). Purified PCR product (400 ng) was denatured (95°C for 5 min) and reannealed by gradually cooling from 95°C to 85°C at −2°C/s and 85°C to 2°C at −0.1°C/s in NEB Buffer 2.1 (New England Biolabs) on a thermocycler. After treatment with T7 Endonuclease I (T7E1; New England Biolabs) for 15 min at 37°C, 250 mM EDTA was added at a final concentration of 6 mM to stop the T7EI reaction, and cleaved DNA fragments were analyzed using High Sensitivity D1000 ScreenTapes on a 2200 TapeStation (Agilent Technologies).

In vitro transcription for sgRNA and mRNA preparation

For the *in vitro* transcription of sgRNA, the PCR template was amplified by the KOD Plus Neo PCR Kit (Toyobo, Cat. No. KOD-401) with a forward primer, which contained a T7 promoter, as shown in **Supplemental Table 2**. Then, sgRNA was in vitro transcribed overnight at 37°C using a MEGAshortscript T7 Transcription Kit (ThermoFisher Cat. No. AM1354) from the PCR template, according to the manufacturer's instructions. RNA purification was performed the following day using a RNeasy MinElute Kit (Qiagen, Cat. No. 74204). For the RNA size-dependent inhibition assay, RNA of less than 500 bp in length were prepared using a MEGAshortscript T7 Transcription Kit, and greater than 500 bp in length were produced using the MEGAscript T7 Transcription Kit (ThermoFisher Cat. No. AM1333). The RNA fragments used are listed in **Supplemental Table 3**.

RNA pull-down assay

For preparation of antibody-coated Dynabeads, 10 μL Dynabeads Protein G (Invitrogen, Cat. No. 10003D) was first incubated in 200 μL PBS + 0.05% Tween with anti-HA-probe (F-7) antibody (Santa Cruz, sc-7392) at a concentration of 1 ng/μL, in a 1.5 mL tube for 30 minutes at room temperature on a rotator. At the end of the incubation, the supernatant was removed using a magnetic stand, and the antibody-coated beads were resuspended in 200 μL PBS + 0.05% Tween for a rinse with gentle pipetting. As a sample, 200 ng rCas9-HA, 50 ng DMD1 sgRNA and 4000 ng total cellular RNA extracted from 293T cells were then combined and made up to 50 μL in a protein buffer containing a final concentration of 20 mM HEPES (pH 7.5), 150 mM KCl, 1 mM DTT and 10% glycerol. As 10% input control for the qPCR analysis downstream, 5 μL of these mixtures were kept aside, and the remaining 45 μL were made up to 300 μL with protein buffer, added to the antibody-coated Dynabeads, and then incubated for 30 minutes at room temperature on a rotator. After removal of the supernatant, the beads bound with RNA/protein were washed 3 times with 200 μL protein buffer and resuspended in 100 μL protein buffer in a new 1.5 mL tube. The supernatant was again removed, and the beads were resuspended in 20 μL of 1 mM Tris (pH 8) and incubated at 65°C for 10 minutes to release the RNA components that would have been bounded to Cas9 protein. The supernatant was collected and used for reverse transcription to produce a cDNA template for qPCR. 5 μL of the supernatant was first incubated at 65°C for 5 minutes to denature any possible secondary structure formed by RNA, then left on ice until use. Reverse transcription was performed using the ReverTra Ace qPCR RT Master Mix (Toyobo, FSQ-201) by mixing 2 μL of 5× RT master mix with 5 μL supernatant, made up to 10 μL with Milli-Q, and then incubated at 37°C for 15 minutes, 50°C for 5 minutes, 98°C for 5 minutes. qPCR was then performed by mixing 1 μL of the reverse-transcribed product with 5 pmol of each of the forward and reverse qPCR primers, 10 μL SYBR Select Master Mix (Applied Biosystems, #4472908), and Milli-Q water to 20 μL total volume. StepOne Real-time PCR system (Applied Biosystems) with standard cycling conditions (Preheating of 95ºC for 10 min, 40 cycles of 95ºC for 15 sec and 60ºC for 1 min) and melting curve conditions described in manufacturer's protocol were used. The amount of sgRNA detected in the samples of the supernatant from the RNA-protein pull-down assay was calculated relative to the positive control sample (sgRNA only with no inhibitory RNA added) using the delta Ct method.

Pluripotency marker flow cytometry analysis

iPSCs and HEK293T cells were trypsinized and suspended in PBS with 5% FBS. Single-cell suspensions were stained for the pluripotent stem cell markers SSEA-4 (Alexa Fluor 647 Mouse anti-SSEA-4, BD Biosciences, Cat. No. 560796) and TRA-1-60 (Alexa Fluor 488 Mouse anti-Human TRA-1-60 Antigen, BD Biosciences, Cat. No. 560173) with an antibody dilution rate of 1:100. No antibody staining and single antibody staining controls were also prepared. After filtering through a 70-μm-pore nylon mesh, stained cells were analyzed by flow cytometry using a BD LSRII Fortessa with BD FACSDiva software (BD Biosciences). Cell debris was excluded from the analysis by using forward- and side-scatter gating. No antibody controls were used to adjust the fluorescence detection range, and single antibody staining controls were used to compensate the fluorescence leakage between Alexa Fluor 488 and 647. The data obtained were analyzed with FlowJo software (Tree Star).

Statistical analysis

For comparison between two groups, we used Welch's t-test calculated by the T.TEST function in Excel software with two tailed, non-paired, unequal variance analysis options. For comparison of three groups or more with a single parameter, one-way ANOVA followed by Tukey's multiple comparisons test in GraphPad Prism software was used. For comparison of several groups with a control, one-way ANOVA followed by Dunnett's multiple comparisons test in Prism software was used. For comparison of several groups with two parameters, two-way ANOVA followed by Sidak’s multiple comparisons test in Prism software was used. $p < 0.05$ was considered statistically significant.

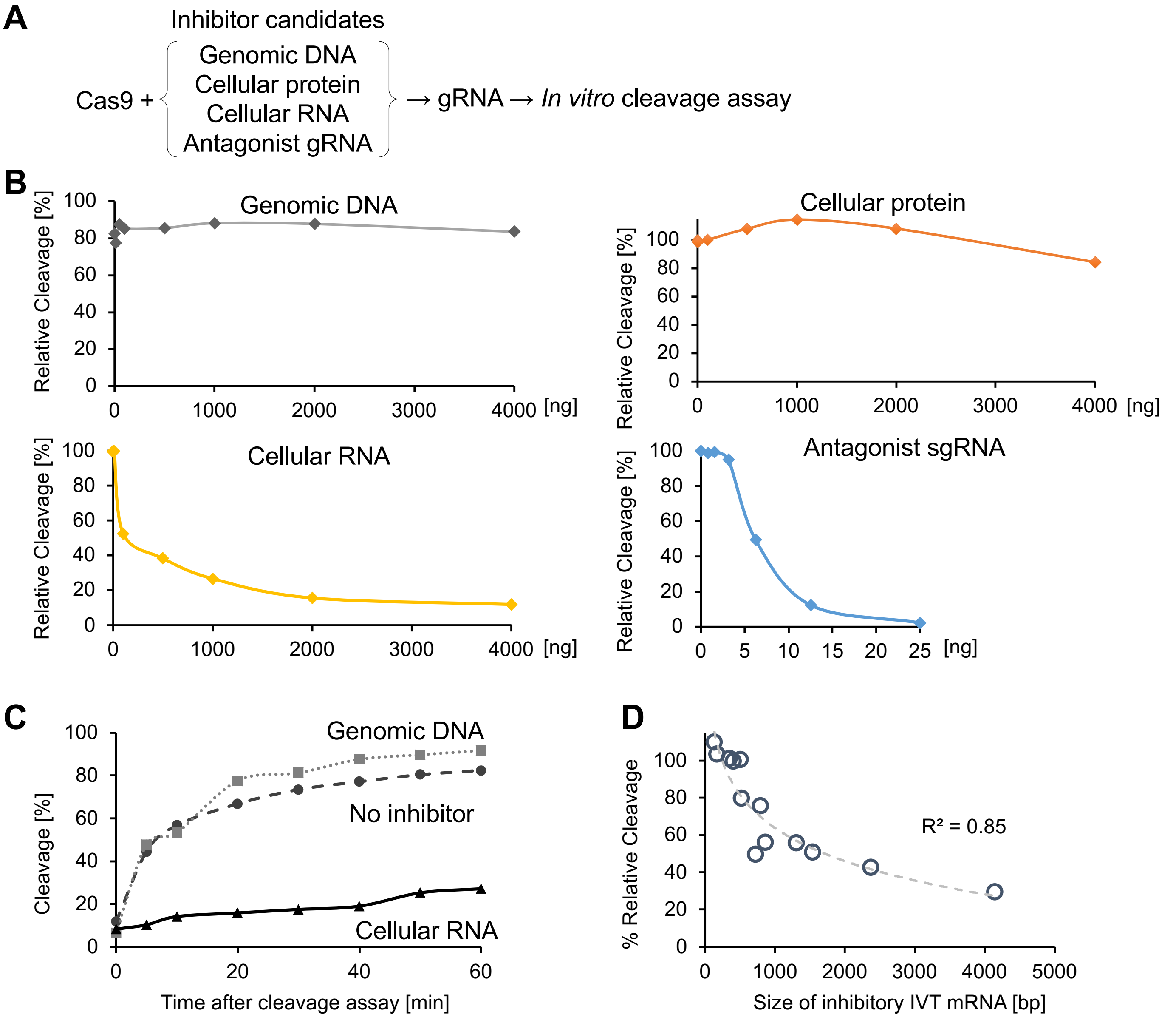

Supplemental Figure 1. Inhibition of Cas9 by cellular RNA and antagonist sgRNA, related to Figure 1.

(A) To examine the inhibitory effect of various cellular components on Cas9 cleavage activity, 50 ng of Cas9 protein was incubated with one of the potential inhibitory factors for 15 minutes at room temperature before the addition of 12.5 ng sgRNA, which targeted human DMD sequence. *In vitro* cleavage reaction was then carried out as described in the Methods section.

(B) To elucidate the effect of various cellular components on Cas9 cleavage activity, we performed an in vitro cleavage assay by first preincubating Cas9 protein with mouse genomic DNA extracted from C2C12 cells (no sgRNA target site), cellular protein and total RNA extracted from HEK293T cells or antagonist sgRNA which targets the HLA-A locus. No apparent inhibition was observed from genomic DNA and cellular proteins, whereas upon preincubation with total RNA relative cleavage was down to 12% at the highest RNA concentration. Also, antagonist sgRNA inhibited relative cleavage activity down to 10% at an equal amount with the targeting DMD sgRNA (12.5 ng), suggesting that sgRNA exchange after binding with Cas9 is not prominent. (C) A time course experiment was performed to elucidate the kinetics of inhibitory effects exerted by genomic DNA and total RNA on Cas9 cleavage activity. We performed an in vitro cleavage assay by first preincubating rCas9 protein with water (negative control), 1,200 ng of genomic DNA or 1,200 ng of total RNA. Samples were collected at 0, 5, 10, 20, 30, 40, 50 and 60 minutes after the beginning of cleavage reaction, and cleavage reaction was stopped by the addition of 1 µL of 0.25 M EDTA and then left on ice until the time course was completed. Results showed that inhibition from total RNA commenced instantaneously and continued throughout the time course.

(D) To examine what type of RNA inhibited on Cas9 cleavage activity, we synthesized various RNAs as shown in Table 2 by *in vitro* transcription and preincubated with Cas9. Results showed that the inhibition on Cas9 cleavage activity was correlated with the size of RNA examined (R^2 = 0.85).

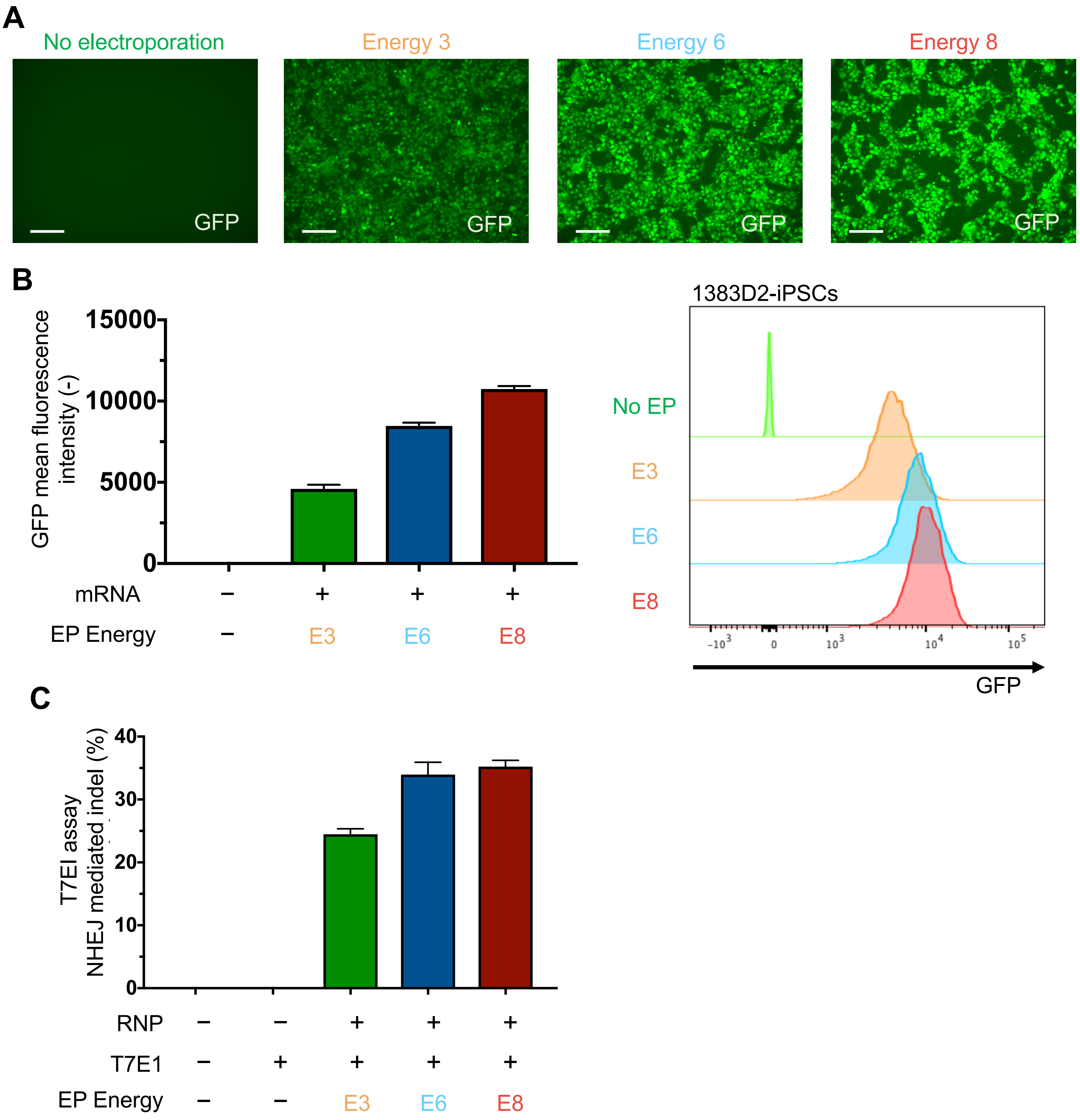

Supplemental Figure 2. Optimization of MaxCyte electroporation, related to Figure 2-5.
(A) 1383D2 human iPSCs were electroporated with GFP mRNA at three electroporation energies (E3, E6, and E8) using MaxCyte ATX. Fluorescence images obtained one day after electroporation are shown. Note that nearly 100% of cells are transfected. There was a modest decrease in cell number at higher energy conditions. Scale bar, 200 µm.
(B) Flow cytometry data of GFP fluorescence intensities are depicted in a bar graph (left panel) or in a half offset overlay plot (right panel) using FlowJo software. The GFP fluorescence signals are presented as mean ± SD from triplicate electroporations.
(C) Pre-complexed Cas9 protein and sgRNA targeting DMD exon 45 was electroporated into 1383D2 iPSCs at three electroporation energies (E3, E6, and E8). Indel efficiencies are presented as mean ± SD from three T7EI assays.

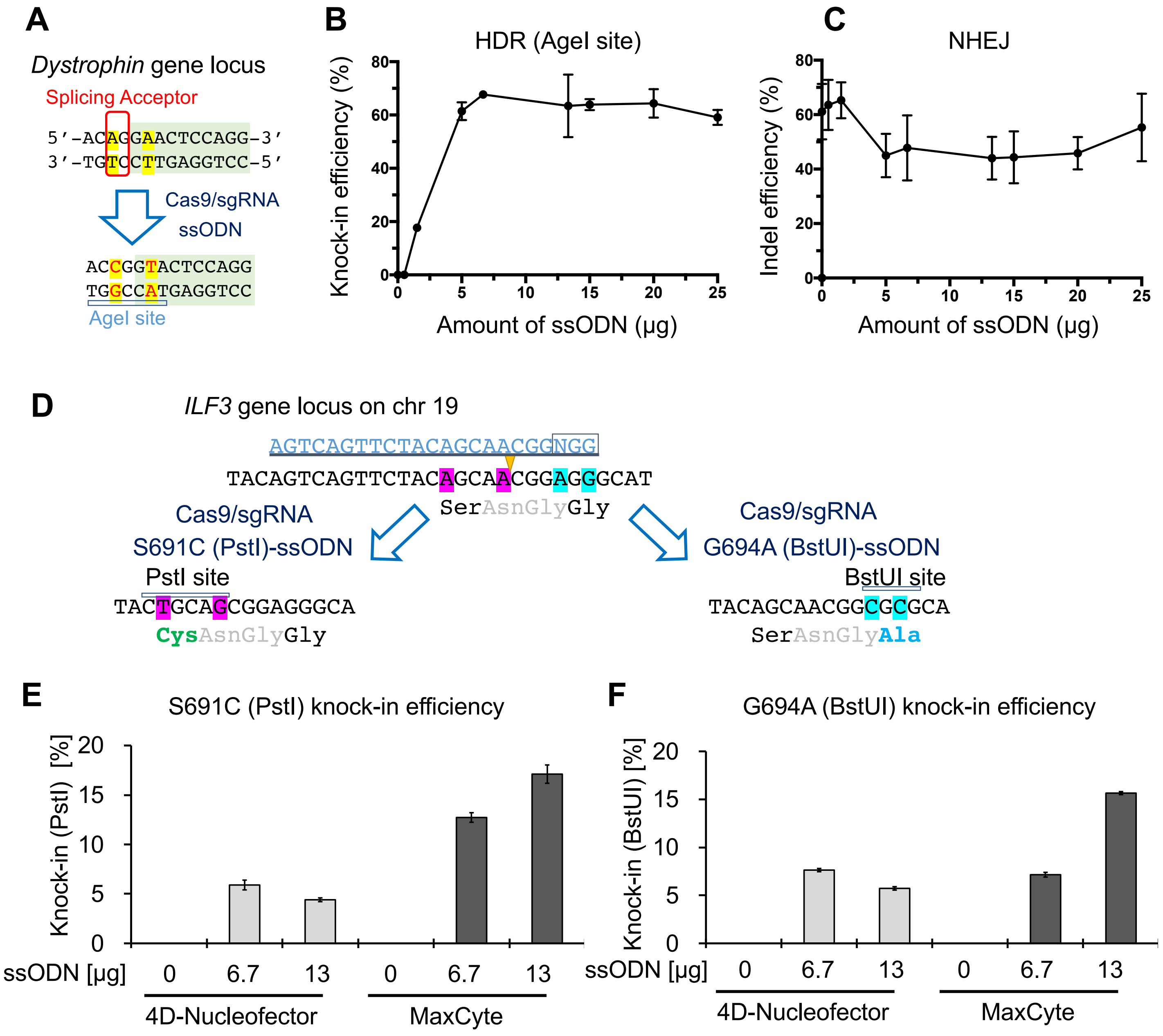

Supplemental Figure 3. Optimization of ssODN and Cas9 RNP mediated targeting, related to Figure 2, 3.

(A) To introduce a mutation at the splicing acceptor of DMD exon 45, we designed an ssODN donor template to replace two nucleotides. Successful knock-in event introduces an AgeI restriction enzyme site (A|CCGGT).

(B) Knock-in efficiency at the *DMD* gene locus in DMD-iPSCs (CiRA00111) was assessed by AgeI restriction enzyme digest, when various amounts of ssODN were electroporated by MaxCyte with energy E8. Scatter plot of ssODN knock-in frequency quantified from three AgeI digestion experiments are represented as mean ± SD (n = 3).

(C) Scatter plot of NHEJ mediated indel frequency measured by three T7EI assays are represented as mean ± SD (n = 3).

(D) Schematic of genomic DNA sequences at the *ILF3* (*NF110*) gene locus on chromosome 19, representing the introduction of Ser671Cys and Gly694Ala substitution with ssODN-mediated knock-in (KI). Successful knock-in sequence generates a PstI or BstUI restriction enzyme site, respectively.

(E) With a healthy donor derived human iPSCs (1383D2 clone), ssODN-mediated genome editing was performed with various ssODN amounts and two electroporation instruments. The knock-in efficiency was evaluated based on the restriction enzyme digestion assay by using PstI enzyme (recognition site: CTGCA|G). PstI knock-in percentages are represented as mean ± SD (n = 3, technical triplicate).

(F) Similar to (B), ssODN-mediated knock-in efficiency was evaluated by the restriction enzyme digestion assay with BstUI enzyme (recognition site: CG|CG). BstUI knock-in efficiencies are represented as mean ± SD (n = 3, technical triplicate).

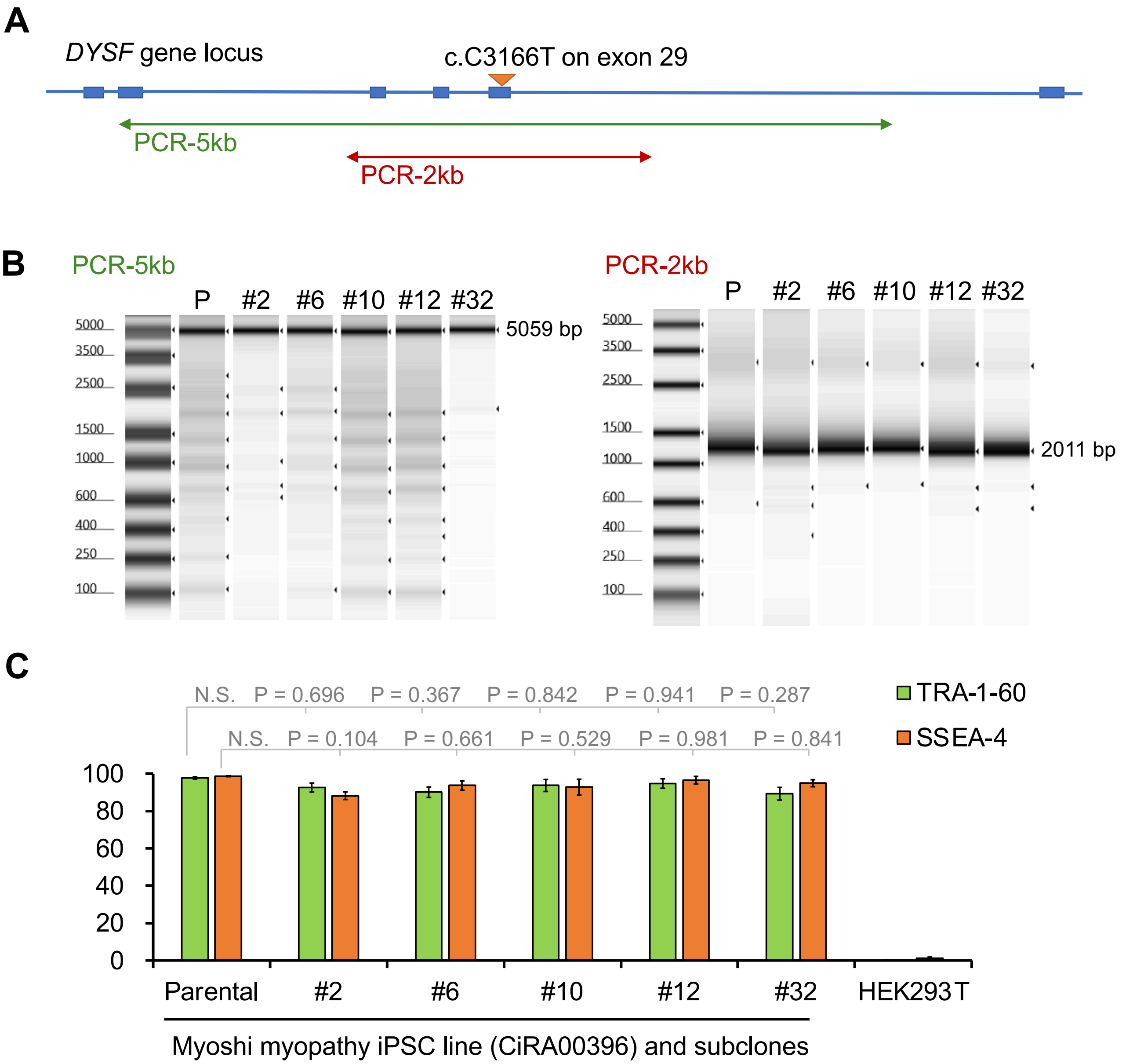

Supplemental Figure 4. Analysis of on-target large deletion and pluripotency, related to Figure 2.
(A) Schematic of the *DYSF* gene locus around the target c.C3166T homozygous mutation site on exon 29. To check whether there an on-target large deletion exists, we designed two primer pairs to amplify 5 kb or 2 kb regions, respectively.
(B) Genomic DNA was extracted from the parental Myoshi myopathy patient iPSC line (CiRA00396) and genome edited subclones that were homozygously (clones #2, #10, #12, and #32) or heterozygously (clone #6) corrected by Cas9/sgRNA using MaxCyte. All 5 clones showed no apparent deletion within the analyzed region.
(C) The above subclones were stained for cell surface TRA-1-60 and SSEA-4 as pluripotent stem cell markers and analyzed by flow cytometer. HEK293T cells were used as negative control. The levels of pluripotency marker expression with the 5 subclones were comparable with the parental iPSC line CiRA00396. Data are represented as mean ± SD (n = 3 experiments). One-way ANOVA followed by Dunnett's test.

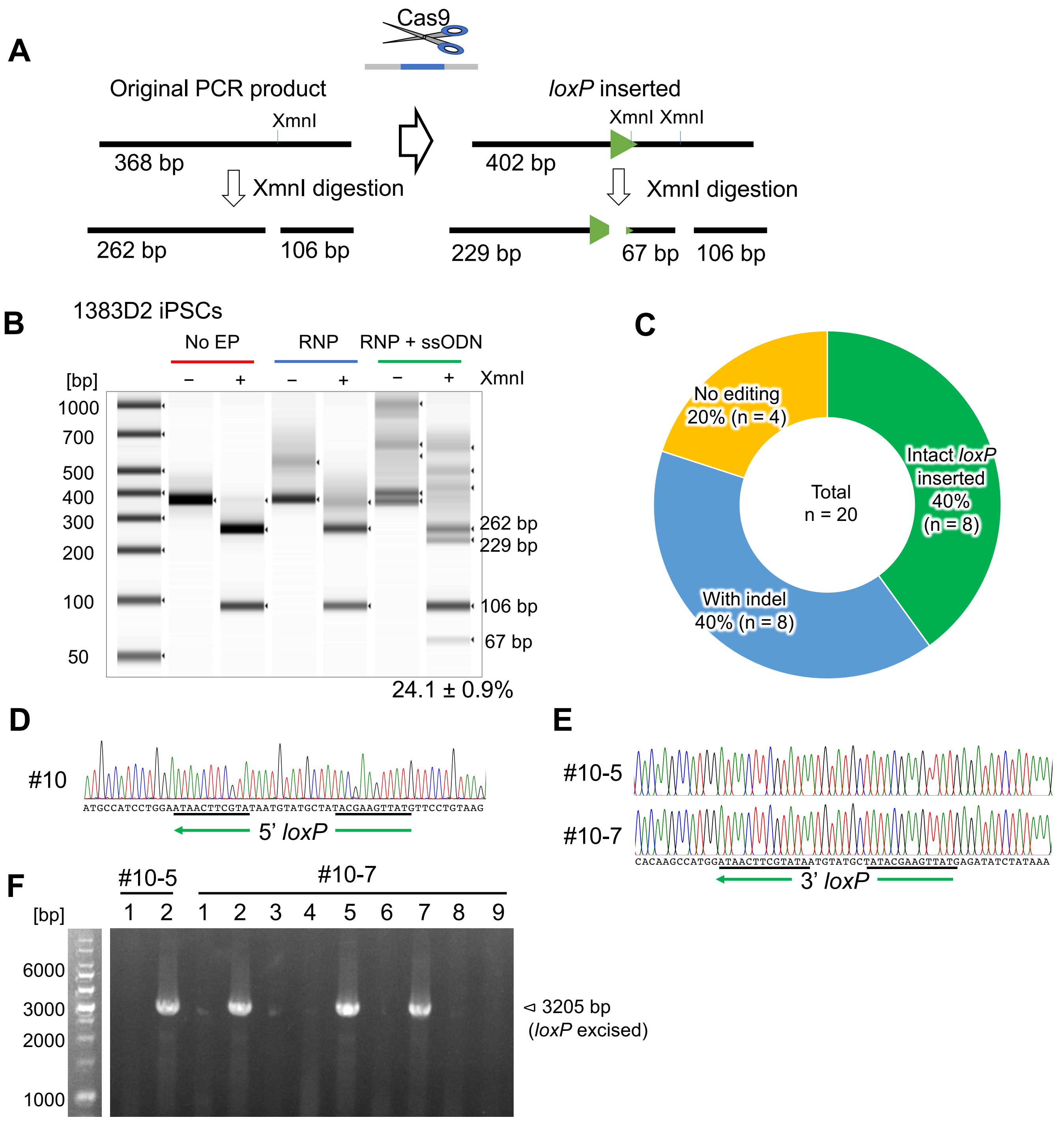

Supplemental Figure 5. Insertion of *loxP* site by ssODN, related to Figure 4.

(A) Schematic to insert a *loxP* sequence at the *DMD* gene locus in 1383D2 iPSCs. Successful insertion of a *loxP* site generates an extra XmnI restriction enzyme site.

(B) TapeStation analysis of PCR products from 1383D2 iPSCs with or without XmnI digestion. With RNP and ssODN electroporation, extra bands with expected sizes can be observed, suggesting the *loxP* sequence is inserted at the target site.

(C) We isolated subclones of 1383D2 iPSCs and genotyped each clone by Sanger sequencing. Pie chart represents 8 subclones out of 20 subclones analyzed have intact *loxP* sequence insertion at the target site.

(D) Sanger sequencing of the 1st *loxP* site in clone #10 (Reverse primer).

(E) Sanger sequencing of the 2nd *loxP* site in clones #10-5 and #10-7 (Reverse primer).

(F) After Cre treatment of clone #10-5 or #10-7, subclones were isolated and genotyped by PCR. Clones #10-5-2, #10-7-2, -5, and -7 showed successful excision of the 342 kb region flanked by the two *loxP* sites.

Supplemental Table 1. PCR and Sanger sequencing primers. Related to Experimental Procedures section.

Name	Sequence
DMD1-qPCR-Fwd	GGTATCTTACAGGAACTCCGT
DMD1-qPCR-Rev	GACTCGGTGCCACTTTTTCAAG
CEL-I-DMD-Fwd	CACCTCTCGTATCCACGATCACTAAG
Ex45 Scr-Rev3 new	TAGTGCCTTTCACCCTGCTTAT
DYSF-in28-L-fwd	AACCTGGCGAGCCCCAT
DYSF-in29-rev	GTGTGATCTGTGCATGCGC
DYSF-ex29-fwd2	ATCACCATCCCCCCGGAG
DYSF-in29-rev2	TGTGGGCATGCAGGTCTG
DYSF_deletion_check_F	GAACGAGACTAAGTTGGCCCTTG
DYSF_deletion_check_R	CAGCTGGTTGTCCAAAAGCC
DYSF_deletion_check2_F	CTGTCTGCCTTGGTCTCTTGCAC
DYSF_deletion_check2_R	CTGGGTGTGATCTGTGTGTGATCTC
T7E1 Fwd new 368bp	CCCTGACACATAAAAGGTGTCTTTCTGT
T7E1 DMD1 Rev	TTCTGTCTGACAGCTGTTTGCAGAC
NF110-ssODN-Fw	TCAGGACCCATGGCTGCCG
NF110-ssODN-Rv	GGGACTGGTAGCCCGAGC
DMDexon55(45-55)check_dir5	CCTCGGGTACACTGAAAGTTATGTG
DMDexon45(45-55)check_rev5	CACCACAGGCTTTAACTTCTGCCG
604B1-HLA-C-be-ex1-fwd	CAATCAGCGTCTCCGCAGT
604B1-HLA-C-ex8-rev	ATGCTAACAGGAACGCAGACA
HLA-C-ex1-fwd	TCCTGCTGCTCTCGGGAG
HLA-C0702-in3-rev	AGGGAGGGCGATATTCCAGT

Supplemental Table 2. Primers used for *in vitro* transcription reaction to prepare sgRNA. Related to Experimental Procedures section.

Name	Sequence
T7-DMD-sgRNA1 fwd	GAAATTAATACGACTCACTATAgggtatcttacaggaactccGTTTTAGAGCTAGAAATAGCAAG
T7-DMD-in55-g3-IVT-fwd	GAAATTAATACGACTCACTATAggactttatagatatctcccaGTTTTAGAGCTAGAAATAGCAAG
T7-DYSF-gRNA2-IVT	GAAATTAATACGACTCACTATAggtactacacacactgacggGTTTTAGAGCTAGAAATAGCAAG
T7-NF110-Ex17-SA2-fwd	GAAATTAATACGACTCACTATAggtcagttctacagcaacggGTTTTAGAGCTAGAAATAGCAAG
T7-HLA-A-0101-ex3-g2-IVT-fwd	GAAATTAATACGACTCACTATAggccgtcgtaggcgtcctgcGTTTTAGAGCTAGAAATAGCAAG
T7-HLA-C7-ex2g2-IVT-fwd	GAAATTAATACGACTCACTATAGgcaggttccgcaggctcactGTTTTAGAGCTAGAAATAGCAAG
sgRNA-+85 rev	AAAGCACCGACTCGGTGCCACTTTTTCAAGTTGATAACGGACTAGCCTTATTTTAACTTGCTATTTCTAGCTCTAAAAC

Supplemental Table 3. RNA fragments for *in vitro* cleavage inhibition assay. Related to Figure S2B.

Source	RNA type	Size (bp)
SpCas9	mRNA	4408
hF8	mRNA	2365
hEEF1G	mRNA	1536
hF8	mRNA	1300
EGFP	mRNA	985
hRPL8	Ribosomal mRNA	856
hF8	mRNA	787
hF8	mRNA	515
hHIST1H2AG	mRNA	498
hF8	mRNA	402
hRPPH1	Non-coding RNA	341
hRNU1-3	Small nuclear RNA	164
FnCas9 sgRNA	Orthogonal sgRNA	154

Supplemental Table 4. Template ssODN sequence for HDR experiments. Related to Experimental Procedures section.

Name	Sequence
DMD1<br>(100-mer)	AAAAAGACATGGGGCTTCATTTTTGTTTTGCCTTTTTGGTATCTTACCGGT<br>ACTCCAGGATGGCATTGGGCAGCGGCAAACTGTTGTCAGAACATTGAAT
DYSF-WT-ssODN<br>(100-mer)	GGAAGCCGAAGCACTGGGTCCCTGCTGAGAAGATGTACTACACACACC<br>GACGGCGGCGCTGGGTGCGCCTGCGCAGGAGGGATCTCAGCCAAATG<br>GAAGC
NF110-BstUI-<br>dPAM-ssODN<br>(100-mer)	GCCTGACCCACTGCCTCCCTGTTTAGGTCAGTTCTACAGCAACGGCGC<br>GCATTCTGGGAATGCCAGTGGCGGTGGCGGCGGGGGCGGTGGTGGCT<br>CCTCC
NF110-PstI-<br>ssODN<br>(100-mer)	GCCTGACCCACTGCCTCCCTGTTTAGGTCAGTTCTACTGCAGCGGAGG<br>GCATTCTGGGAATGCCAGTGGCGGTGGCGGCGGGGGCGGTGGTGGCT<br>CCTCC
DMD1+loxP-<br>ssODN<br>(134-mer)	AAGACATGGGGCTTCATTTTTGTTTTGCCTTTTTGGTATCTTACAGGAACA<br>TAACTTCGTATAGCATACATTATACGAAGTTATTCCAGGATGGCATTGGGC<br>AGCGGCAAACTGTTGTCAGAACATTGAATGCA
DMD-in55-<br>g3+loxP-ssODN<br>(134-mer)	TCATTTGGAGGTAATTTGTTTGGAACAGTATCAGACTTTATAGATATCTCAT<br>AACTTCGTATAGCATACATTATACGAAGTTATCCATGGCTTGTGATAGAATA<br>TAAGGGCAATGCAAATGTAGAGTTTTTTGC
C0702-1bp-mut-<br>AS<br>(100-mer)	CGGGGTCACTCACCGTCCTCGCTCTGGTTGTAGTAGCCGCGCAGGTTC<br>CGCAGaCTCACTCGGTCAGCCTGTGCCTGGCGCTTGTACTTCTGTGTCT<br>CCC
C0702-2bp-mut-<br>AS<br>(100-mer)	CGGGGTCACTCACCGTCCTCGCTCTGGTTGTAGTAGCCGCGCAGcTTC<br>CGCAGGtTCACTCGGTCAGCCTGTGCCTGGCGCTTGTACTTCTGTGTCT<br>CCC
